# Toll-like receptor 2/6-stimulated HMC-1 mast cells promote keratinocyte migration in wound healing

**DOI:** 10.1371/journal.pone.0317766

**Published:** 2025-01-17

**Authors:** Jiyun Kwon, Kyung-Ah Cho, So-Youn Woo

**Affiliations:** Departments of Microbiology, College of Medicine, Ewha Womans University, Seoul, Korea; Universidade Federal do Rio de Janeiro, BRAZIL

## Abstract

Mast cells, immune sentinels that respond to various stimuli in barrier organs, provide defense by expressing pattern recognition receptors, such as Toll-like receptors (TLRs). They may affect inflammatory responses and wound healing. Here, we investigated the effect of TLR2/6-stimulated mast cells on wound healing in keratinocytes. The human mast cell line HMC-1 was treated with the TLR2/6 agonist FSL-1, and the conditioned medium (CM) was collected from untreated cells (HMC-1 CM) and FSL-1-stimulated cells (FSL-1-HMC-1 CM). Cell migration was evaluated in keratinocyte cells (HaCaT) treated with HMC-1 CM and FSL-1-HMC-1 CM via scratch and Transwell assays. Mice were treated with HMC-1 CM, FSL-1-HMC-1 CM, and FSL-1. Wound closure was measured, and tissue regeneration was assessed using hematoxylin and eosin staining. Growth factor expression levels were evaluated to identify the factors affecting wound healing. The tryptase inhibitor APC 366 was treated with HMC-1 CM and FSL-1-HMC-1 CM in a scratch assay. This study revealed that HMC-1 CM affected HaCaT cell migration, which was facilitated by FSL-1-HMC-1 CM. HMC-1 CM promoted tryptase-dependent HaCaT migration. Moreover, FSL-1-HMC-1 CM enhanced wound healing in C57BL/6J mice *in vivo*. Our findings indicated that TLR2/6-stimulated mast cells contributed to skin homeostasis by promoting tryptase-dependent wound healing.

## Introduction

Mast cells, which are derived from hematopoietic stem cells, are essential immune effectors involved in innate and adaptive immune responses. They affect proinflammatory reactions by releasing proinflammatory and angiogenic factors, including TNF-α, IL-6, vascular endothelial growth factors (VEGFs), stem cell factor (SCF), matrix-degrading enzymes, IL-9, tryptase, histamine, granzyme B, and reactive oxygen species. They cause bronchial asthma, allergic rhinitis, urticaria, food allergy, atopic dermatitis, and angioedema by participating in IgE-mediated allergic reactions [1]. They also express pattern recognition receptors, such as NOD-like receptors, C-type lectin receptors, and Toll-like receptors (TLRs) [2]. TLRs detect microorganism-associated molecular patterns (MAMPs) and enhance responses through the IgE receptor FcεRI [3, 4]. Through this activation, they can regulate immune responses to internal and external factors, including allergen-specific IgE [5, 6]

Mast cells are mainly found in the border regions of vascularized tissues such as intestines, airway mucosa, connective tissue, and skin [7]. Skin mast cells play a key defense role against invading endogenous stimuli (such as B-defensins, LL37, substance P, VIP, CRH, somatostatin, IL-1, IL-33, IL-9, and tryptase) and exogenous stimuli (such as drugs, allergens, food, alcohol, bee stings, stress, physical stimuli, and infections); they do so by participating in degranulation (histamine, proteases, tryptase, matrix metalloproteinase, and heparin release), producing lipid mediators (prostaglandin D2, leukotriene C4, and platelet-activating factor), and producing cytokines and chemokines (TNF-α, IL-6, IL-8, IL-33, INF-γ, VEGF, epidermal growth factor, fibroblast growth factor, and granulocyte-macrophage colony-stimulating factor) [8, 9].

Keratinocytes form a protective epidermal barrier composed of a multilayered epithelium that plays essential wound healing and skin repair roles. They are implicated in epithelialization through which they restore the epidermal barrier by migrating, proliferating, and differentiating [10]. For re-epithelialization during wound healing, keratinocytes proliferate, differentiate, migrate, and promote angiogenesis; they also experience crosstalk with immune cells via cytokines and chemokines [11, 12].

Increased vascular permeability and vessel dilation caused by mast cell-derived histamine, VEGF, IL-6, and IL-8 stimulate the migration of inflammatory cells to wound sites. In the proliferative stage of wound healing, keratinocyte-released SCF attracts mast cells to the epidermis [7]. In the present study, we investigated the role of mast cells in early wound healing and tissue regeneration, especially under TLR stimulation, by using HaCaT cells and a mouse model.

## Materials and methods

### Cell culture

HaCaT and HMC-1 were cultured in DMEM and IMDM, respectively, which were supplemented with 10% fetal bovine serum (FBS, WELGENE, South Korea) and penicillin (100 U/mL)/streptomycin (100 μg/mL; Capricorn Scientific, Ebsdorfergrund, Germany). The human monocyte cell line THP-1 was cultured in RPMI 1640 (WELGENE) supplemented with 10% FBS, penicillin (100 U/mL)/streptomycin (100 μg/mL), and 50 μM 2-mercaptoethanol. All cell lines were cultured in a humidified incubator with 5% CO_2_ at 37°C.

### Conditioned medium (CM) preparation

HMC-1 cells were cultured in 100 mm cell culture plates to a confluence of 90%–95% to prepare the CM. The culture medium was then changed to serum-free IMDM (10 ml). After 48 h of culture, the medium was collected, transferred into a Macrosep Advance centrifugal device with an Omega membrane (Pall, Port Washington, NY, USA) with a membrane cut-off of 3K, and centrifuged at 5,000 × *g* for 1 h (Sorvall LYNX4000, ThermoFisher Scientific, Waltham, MA, USA) at 4°C. It was generally concentrated to a 5-fold concentration. HMC-1 cells cultured in FBS-containing IMDM were also treated for 24 h with human TLR 1–9 agonists (InvivoGen, San Diego, CA, USA). Then, the medium was changed to serum-free IMDM for 48 h and collected as described above. For mouse skin application, the CM was concentrated using an ultra-centrifugal filter (Millipore, Burlington, MA, USA) and stored at −80°C until use.

### Scratch migration assays

HaCaT cells were seeded into 12-well plates and cultured to 100% confluence. The monolayer was scratched using a cell scraper, and cell debris was removed by gently rinsing with PBS twice. HaCaT cells were cultured in serum-free IMDM, HMC-1 CM, and FSL-1-HMC-1 CM (100 μl) for 24 and 48 h. The cell proliferation inhibitor mitomycin C (Roche, Basel, Switzerland) was used at a concentration of 10 μg/ml. The cells were pre-treated with mitomycin C for 2 h to inhibit proliferation; then, a scratch assay was performed. The tryptase inhibitor APC 366 (Sigma–Aldrich, St. Louis, MO, USA) was simultaneously administered at 20 μg/ml, and its efficacy was evaluated. All experiments were conducted in triplicate.

### Transwell migration assays

Transwell migration assays were performed using Transwell® 24-well permeable support plates (pore size: 8.0 μm, Corning, NY, USA). HaCaT cells (2×10^5^) were seeded in the upper chamber with DMEM (50 μl) containing 2% FBS, and treated with 50 μl of CM or serum-free IMDM. The lower chamber was filled with DMEM supplemented with 2% FBS (600 μl). The cells were incubated at 37°C for 24 and 48 h. The migrated cells were stained with 0.2% crystal violet (MERCK, Rahway, NJ, USA) and quantified on ImageJ (https://imagej.net/ij/).

### Wound healing mouse model

Nine-week-old female C57BL/6J mice (EWHA MED IACUC 22-008-t) were purchased from Raonbio (Gyeonggi-do, South Korea). A 200 μl mixture of 6% Zoletyl, 4% Rumphen, and 90% PBS was administered to each mouse. The mice were anesthetized through intraperitoneal (IP) injection. Dermal excision wounds (diameter: about 6 mm) were made using scissors on the backsides under anesthesia. After 1 and 3 days, the wounds were treated with 60 μl of serum-free IMDM, HMC-1 CM, FSL-1-HMC-1 CM, and FSL-1 (100 μg/ml), and the wound area was measured after 1, 3, 6, and 12 days. The mice were housed with free access to food and water under pathogen-free conditions at 21–23°C, 51%–54% humidity, and a 12 h light–dark cycle.

### Human growth factor antibody array analysis

The RayBio Human Growth Factor Array C1 (Ray Biotech, Peachtree Corners, GA, USA) was used to analyze HMC-1 CM and FSL-1-HMC-1 CM in accordance with the manufacturer’s protocol. Briefly, HMC-1 CM and FSL-1-HMC-1 CM were used at the same concentrations. After the membrane was blocked, the array membrane was incubated with HMC-1 CM and FSL-1 CM for 24 h, washed, incubated with a biotinylated antibody for 2 h, washed again, treated with HRP–streptavidin, and imaged on an ImageQuant LAS 3000 system (GE Healthcare, Little Chalfont, UK).

### Histology

Mouse back skin samples were fixed with 4% formaldehyde, paraffin-embedded, sectioned at a 5 μM thickness, placed on slides, and stained using hematoxylin and eosin (H&E). Tissue reconstruction was then evaluated at a 100× magnification by using an OLYMPUS BX50 microscope.

### Reverse transcription-PCR (RT-PCR)

Total RNA was extracted using a RNeasy Mini kit (QIAGEN, Hilden, Germany) and retrotranscribed into complementary DNA by using a Transcription Master Mix (ELPIS-BIOTECH, Daejeon, South Korea) in accordance with the manufacturer’s protocol. Briefly, RNA was denatured at 70°C for 5 minutes, mixed with 4 μl of the Reverse Transcription Master Premix, and incubated at 42°C for 1 h. It was further incubated at 94°C for 5 minutes. RT-PCR analysis was performed using Maxime™ PCR PreMix (iNtRON, Gyeonggi-do, South Korea). The primers are shown in Table 1. The following program was performed twice: 30 s at 94°C (denaturation), 30 s at 62°C (annealing), and 30 s at 72°C (extension) for 35 cycles. Relative fold expression was examined using DNA gel electrophoresis.

**Table 1:** List of the primers used in this study.

Target gene	Primer Sequence (5′–3′)
*Gapdh*	F: GGTAAAGTGGATATTGTTGCCATCAATG
	R: GGAGGGATCTCGCTCCTGGAAGATGGTG
*Tlr1*	F: TGAACCTCAAGCACTTGGACC
	R: CCCATAAGTCTCTCCTAAGACCA
*Tlr2*	F: ATCCTCCAATCAGGCTTCTCT
	R: GGACAGGTCAAGGCTTTTTACA
*Tlr3*	F: CAAACACAAGCATTCGGAATCTG
	R: AAGGAATCGTTACCAACCACATT
*Tlr4*	F: AGACCTGTCCCTGAACCCTAT
	R: CGATGGACTTCTAAACCAGCCA
*Tlr5*	F: CGATGGACTTCTAAACCAGCCA
	R: GGTTGTCAAGTCCGTAAAATGC
*Tlr6*	F: TGAATGCAAAAACCCTTCACCT
	R: CCAAGTCGTTTCTATGTGGTTGA
*Tlr7*	F: CACATACCAGACATCTCCCCA
	R: CCCAGTGGAATAGGTACACAGTT
*Tlr8*	F: AACTGCCAAGCTCCCTACG
	R: CAAGGCACGCATGGAAATGG
*Tlr9*	F: CTGCCACATGACCATCGAG
	R: GGACAGGGATATGAGGGATTTGG
*IGFBP2*	F: GACAATGGCGATGACCACTCA
	R: CAGCTCCTTCATACCCGACTT
*SCFR*	F: CGTTCTGCTCCTACTGCTTCG
	R: CCCACGCGGACTATTAAGTCT
*PDGFA*	F: GCAAGACCAGGACGGTCATTT
	R: GGCACTTGACACTGCTCGT
*VEGFA*	F: AGGGCAGAATCATCACGAAGT
	R: AGGGTCTCGATTGGATGGCA

F: forward, R: reverse

### RT-qPCR

RNA was extracted and retrotranscribed as described above. Next, master mixes containing reverse and forward primers (Table 1) and the SensiMix™ SYBR® Hi-ROX mix (Bioline, London, UK) were prepared and analyzed using MicroAmp™ Optical 96-well reaction plates (Applied Biosystems, Foster City, CA, USA).

### ELISA

Secreted tryptase levels were measured in HMC-1 CM, FSL-1-HMC-1 CM, HaCaT CM, THP-1 CM, and serum-free IMDM using a human tryptase ELISA kit (Innovative Research Inc., Novi, MI, USA) in accordance with the manufacturer’s protocol. Biotinylated polyclonal antibodies were added to HMC-1 CM, FSL-1-HMC-1 CM, HaCaT CM, and THP-1 CM. After they were washed with PBS, an avidin–biotin–peroxidase complex was added, washed again, and added with the HRP substrate (TMB). Absorbance was read on a microplate reader (Molecular Devices, San Jose, CA. USA).

### Statistical analyses

Data were statistically analyzed using t-test and one-way ANOVA. All data are presented as mean ± standard error of the mean in GraphPad Prism version 10.2.1 (GraphPad Software Inc., La Jolla, CA, USA). Results with P < 0.05 were considered statistically significant.

## Results

### HMC-1 CM affects HaCaT cell migration

**Fig 1:**
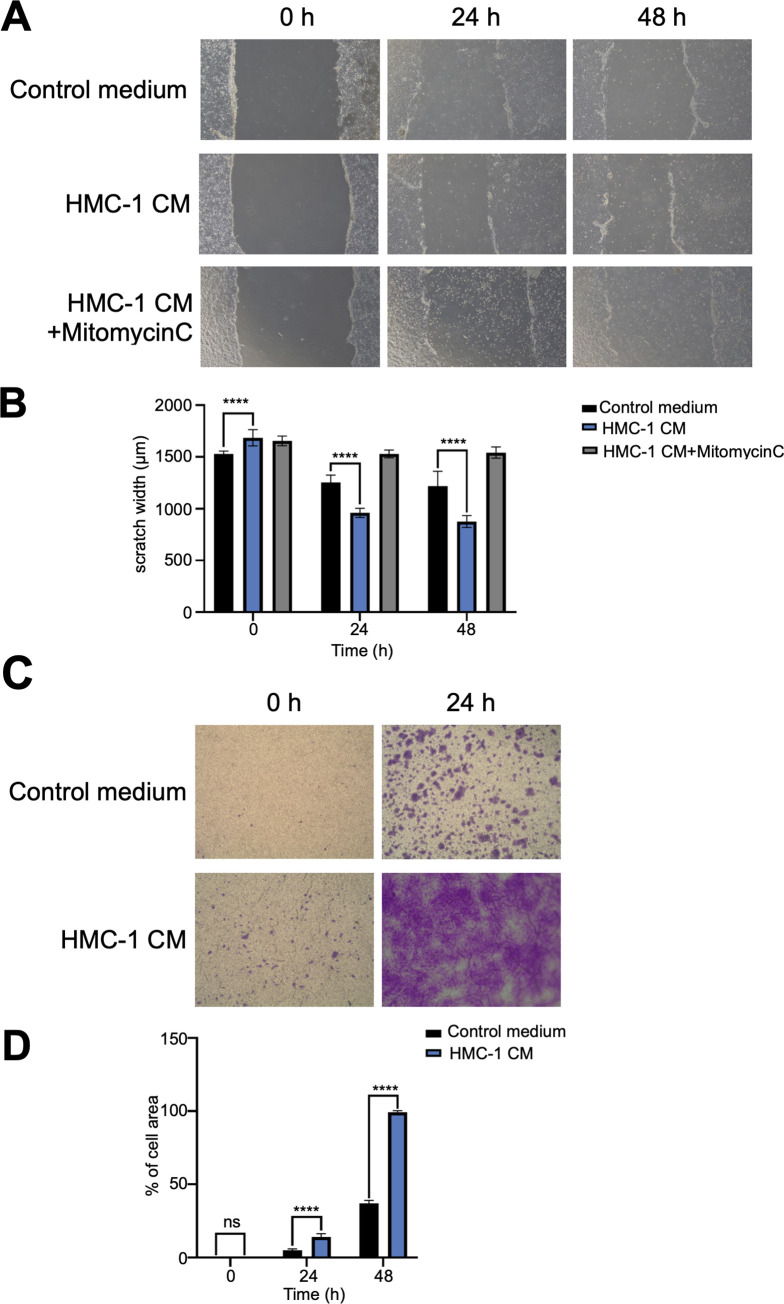
Effects of HMC-1 cells on keratinocyte movement. (A) At every 24 h time point, HMC-1 CM-treated HaCaT cells filled the wound area faster than the vehicle-treated control cells did (n = 3). (B) At the 24- and 48 h time points, scratch assays revealed significant differences (*** and **** indicate P < 0.0001 and < 0.0001, respectively). (C) Transwell assays of HaCaT migration and invasion after incubation for 24 and 48 h. Upper chambers contained HMC-1 CM (n = 3). (D) Transwell assays showed significant differences at the 24- and 48 h time points.

To evaluate the effect of HMC-1 CM on HaCaT cell migration, we treated them with HMC-1 CM and serum-free IMDM for 24 and 48 h; then we performed a scratch migration assay (Fig 1A). We found that wound closure was significantly faster in HMC-1 CM than in serum-free IMDM (control) at the 24 and 48 h time points (Fig 1B). Notably, in the presence of HMC-1 CM, HaCaT cells migrated more in the first 24 h. We obtained similar results in Transwell assays (Fig 1C), as revealed by the visualization and quantification (ImageJ) of the migrated HaCaT cells through crystal violet staining (Fig 1D).

### FSL-1-HMC-1 CM promotes HaCaT cell migration

**Fig 2:**
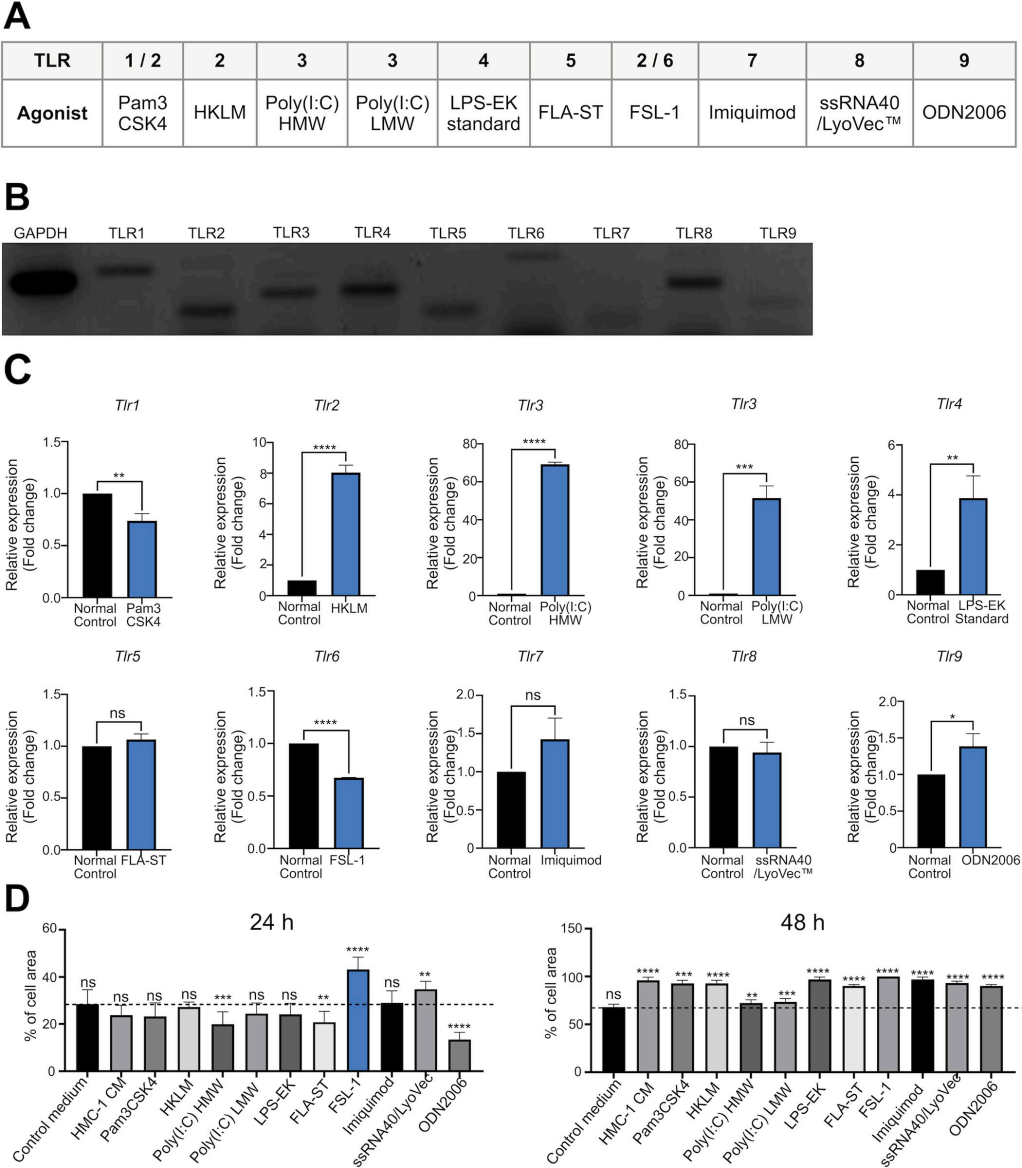
Effects of TLR agonist-stimulated mast cells. (A) A comprehensive table shows Toll-like receptors (TLRs) and their agonists. TLRs and their specific agonists provide valuable insights into the signaling pathways they activate. (B) Reverse transcription PCR analysis of TLR gene expression in HMC-1 cells. (C) Reverse transcription-quantitative PCR analysis of the expression of TLRs 1–9 in TLR agonist-treated mast cells (n = 3). Statistical analysis revealed significant changes in TLR expression (*, **, ***, and **** indicate P < 0.05, < 0.01, < 0.0001, and < 0.0001, respectively. (D) The effect of HMC-1 CM obtained with or without TLR agonist stimulation on HaCaT cell migration was assessed using wound closure measurements (n = 10). Statistical analysis revealed significant differences in wound closure rates (*, **, ***, and **** indicate P < 0.05, < 0.01, < 0.0001, and < 0.000, respectively).

Mast cells participate in allergic and inflammatory responses through their reaction to external stimuli and activation of various receptors, including TLRs, which detect pathogen-associated molecule patterns; as a result, granules are released by mast cells, proinflammatory responses are triggered, and immune reactions are activated. Agonists for TLRs 1 through 9 have been commercially established (Fig 2A). We confirmed the constitutive expression of TLRs (Fig 2B). Next, we aimed to determine whether the changes in the expression of the corresponding receptors of the TLR agonists listed in Fig 2A were associated with the effects of CM from the activated mast cells. RT-qPCR revealed the changes in the expression of TLR1 to TLR9 in HMC-1 cells (Fig 2C). Furthermore, we assessed the effect of TLR agonists on HMC-1 cells by treating HMC-1 CM stimulated with each TLR agonist and exposing on HaCaT cells for 24 and 48 h (Fig 2D). We showed that HaCaT cell migration was promoted more effectively by HMC-1 CM stimulated with the TLR 2/6 agonist FSL-1 than by other TLR-stimulated HMC-1 CM for 24 h.

### FSL-1-HMC-1 CM promotes early wound healing *in vivo*

**Fig 3:**
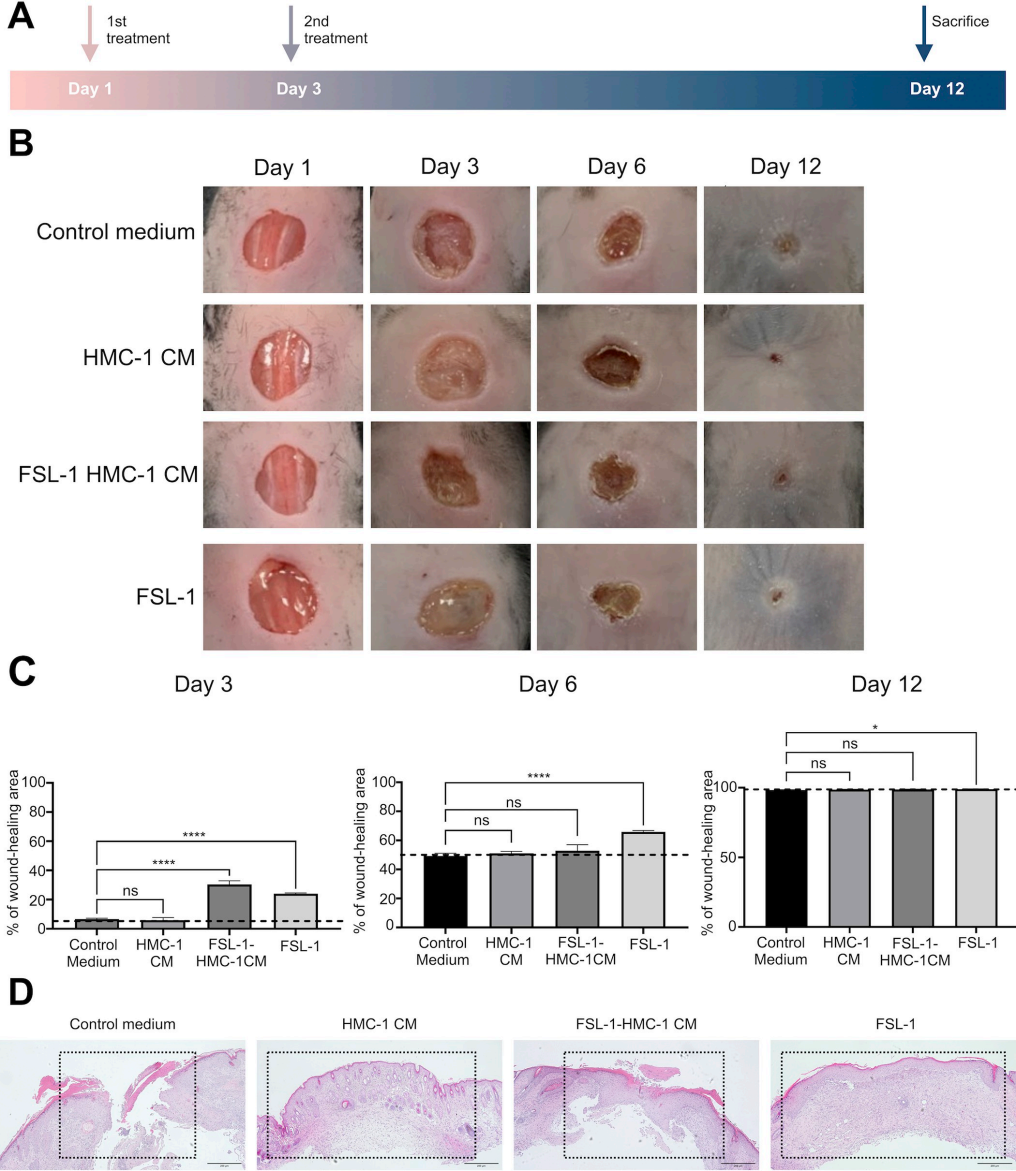
Effects of treatment with HMC-1 CM and FSL-1-HMC-1 CM. (A) The *in vivo* wound-healing experimental plan. Mice were categorized into vehicle, HMC-1 CM, FSL-1-HMC-1 CM, and FSL-1 groups. (B) The wound area was examined through imaging on days 0, 3, 6, and 12. The effects of various treatments on wound healing were qualitatively analyzed by imaging the wound closure progression during the experiment. (C) Wound closure was expressed as the percentage of area closure (n = 3). (* and **** indicate P < 0.05 and < 0.0001, respectively). (D) H&E wound healing analysis on day 12 (magnification: 100X).

Because FSL-1-treated HMC-1 CM (FSL-1-HMC-1 CM) enhanced HaCaT migration, the *in vivo* wound healing effects of HMC-1 CM, FSL-1-HMC-1 CM, and FSL-1 were evaluated using the experimental timeline shown in Fig 3A. To this end, a 6 mm surgical incision was made on the dorsal areas of nine-week-old female C57BL/6J mice; then, serum-free IMDM, HMC-1 CM, FSL-1-HMC-1 CM, and FSL-1 (60 μl; 100 μg/ml) were applied after 1 and 3 days. Wound area was monitored through imaging on days 1, 3, 6, and 12 (Fig 3A). The results showed that wound closure (the percentage of closed area) was significantly different between the FSL-1-HMC-1 CM-treated group and the other groups (Fig 3B and 3C). On day 3, closure in the FSL-1-HMC-1 CM- and FSL-1-treated groups was approximately 3- and 2-fold higher than that in the serum-free IMDM-treated group, respectively. On days 6 and 12, wound area closure was the most rapid in the FSL-1 treatment groups. In terms of HMC-1 CM-treated groups, the results on day 3 indicated that the wound surface in the CM-treated group was nearly obscured by a translucent membrane compared with that in the control group. Although this observation was not directly correlated with a reduction in the actual wound size, it indicated that keratinocyte migration was occurring actively. Histology can reveal cellular- and tissue-level changes associated with wound healing; thus, the effects of treatment on wound tissue morphology and remodeling can be assessed. Here, healing progression was evaluated via H&E analysis (Fig 3D). The results revealed noticeable FSL-1-HMC-1 CM effects on mouse wound healing.

### FSL-1-stimulated HMC-1 cells exhibited growth factor and cytokine changes

**Fig 4:**
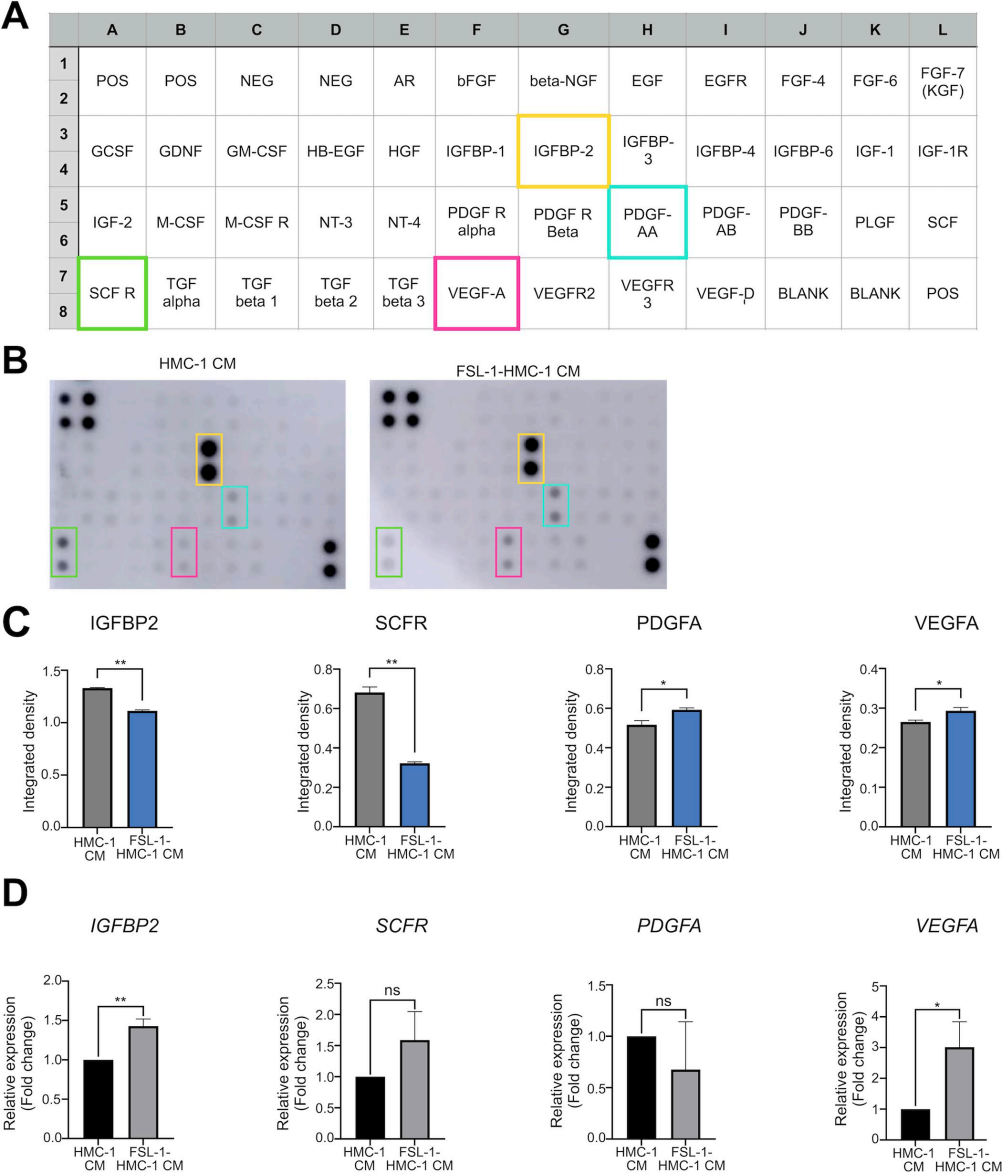
Growth factor cytokines in HMC-1 and FSL-1-HMC-1 CM. (A) A comprehensive table detailing the arrangement of the growth factor cytokines included in the cytokine array assay. (B) Cytokine array analysis of HMC-1 CM and FSL-1-treated HMC-1 CM. (C) Each cytokine array sample was analyzed in duplicate spots. (D) Reverse transcription-quantitative PCR analysis of the expression of growth factors, including IGFBP2 (black), SCFR (green), PDGFA (blue), and VEGF-A (red), after cytokine array analysis (n = 4). Statistical analysis revealed significant differences in the expression levels of these growth factors in HMC-1 CM vs. FSL-1-HMC-1 CM conditions (* and ** indicate P < 0.05 and < 0.01, respectively).

Next, we examined which growth factors or cytokines enhanced mouse wound closure. To determine if growth factor or cytokine secretion was elevated in HMC-1 CM and FSL-1-HMC-1 CM, we used a cytokine array analysis. The table showed the arrangement of the membrane by using double spots for the cytokine array (Fig 4A). We revealed that HMC-1 CM had higher insulin-like growth factor binding protein 2 (IGFBP-2) and stem cell factor receptor (SCF-R) levels. Furthermore, FSL-1-HMC-1 CM increased platelet-derived growth factor AA (PDGF-AA) and VEGF-A levels (Fig 4B and 4C). Although FSL-1-HMC-1 CM was associated with enhanced HaCaT cell migration, its cytokine and growth factor levels did not differ significantly from those of HMC-1 CM. However, cytokine expression analysis using RT-qPCR revealed that the mRNA levels of IGFBP-2 and VEGF-A increased (Fig 4D).

### HMC-1 CM and FSL-1-HMC-1 CM promote tryptase-dependent HaCaT migration

**Fig 5:**
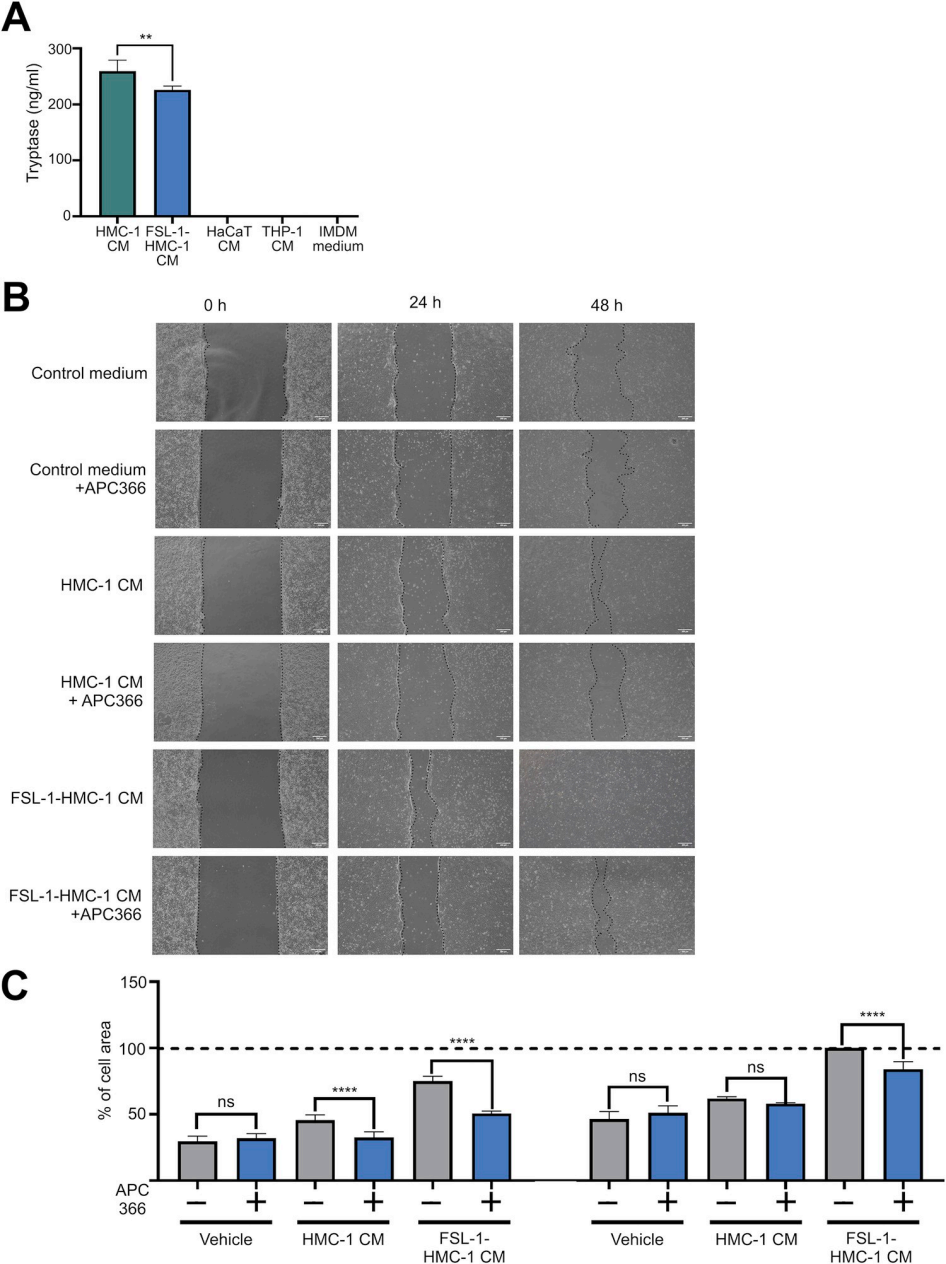
Inhibition of HaCaT cell migration by APC 366. (A) Tryptase secretion levels in HMC-1 CM, FSL-1-HMC-1 CM, HaCaT CM, and THP-1 CM were evaluated using an enzyme-linked immunosorbent assay (n = 3). As a control, basal tryptase secretion was assessed in IMEM (** indicates P < 0.001). (B) The effect of APC 366, a specific tryptase inhibitor, on HaCaT cell migration was evaluated and compared with that on untreated cells. (C) After 24 h (left, n = 10), APC 366 inhibited the migration of HaCaT cells cultured in HMC-1 CM and FSL-1-HMC-1 CM; significant inhibition was observed in FSL-1-HMC-1 CM after 48 h (right, n = 10). **** indicates P < 0.0001.

Because various factors may affect wound healing, we investigated various mast cell-secreted factors, including tryptase, via ELISA. We revealed that HMC-1 CM and FSL-1-HMC-1 CM contained high tryptase levels; THP-1 and HaCaT cells expressed tryptase poorly (Fig 5A). These results indicated that the amount of tryptase secreted by mast cells was more abundant than that secreted by the other cell lines. Moreover, treatment with APC 366 reduced wound healing, indicating that wound healing was tryptase dependent. This inhibitory effect was particularly significant in the groups treated with HMC-1 CM and FSL-1-HMC-1 CM for 24 h (Fig 5B and 5C).

## Discussion

This study found that mast cell CM affected keratinocyte migration and enhanced wound closure *in vivo*; therefore, mast cell factors potentially improved early wound healing and tissue regeneration. Although mast cells are mainly associated with allergy and immune response [13], emerging evidence indicates that they are also involved in inflammation, tissue regeneration, cancer development and progression, and immune regulation. Stimulated mast cells release various factors, including cytokines, histamine, and proteases [14]. Among them, tryptase protects against stimuli such as bacterial eradication and vasodilation by triggering adaptive responses and modulating inflammation [15, 16]. For example, through the tryptase–PAR-2 pathway, skin-resident human mast cells instruct keratinocytes to produce thymic stromal lymphopoietin (TSLP) [17]. Therefore, mast cell tryptase could influence keratinocyte function by regulating organ functions, recruiting various immune cell types, and stimulating defense mechanisms. The interaction between mast cells and keratinocytes might play a pivotal role in maintaining skin homeostasis, regulating inflammation, and facilitating wound healing.

As shown in Fig 2D, in HaCaT cell migration assays, the wound closure rate was highest when HMC-1 cells were treated with the TLR2/6 agonist FSL-1 for 24 hours, compared to other TLR agonists. However, as shown in Fig 5A, ELISA results revealed that treatment with the TLR2/6 agonist FSL-1 for 24 hours resulted in lower tryptase levels compared to untreated HMC-1 cells. Low-dose tryptase promotes wound healing by considerably enhancing fibroblast and macrophage migration, attachment, and proliferation [18]. As such, FSL-1-treated HMC-1 CM could still enhance HaCaT cell migration although tryptase levels decreased. Because mast cells respond to infectious agents or damaged cells promptly, they recognize pathogen-associated molecular patterns or damage-associated molecular patterns via pattern recognition receptors. Among various pattern recognition receptors, TLRs 1–13 are expressed by mast cells [2]. Here, we found that treatment with CM from HMC-1 enhanced HaCaT cell migration *in vitro*. Additionally, the effect of CM from FSL-1-treated HMC-1 cells on HaCaT cell migration was more significant than that of CM from HMC-1 cells treated with other TLR agonists, especially after 24 h (Fig 2D). Furthermore, FSL-1-HMC-1 CM and FSL-1 significantly promoted wound healing and tissue reconstruction *in vivo* (Fig 3). These observations implied that TLR2/6 activation could trigger the release of various proteins alongside tryptase; therefore, inflammatory responses could be collectively amplified, and wound healing could be facilitated. However, the crucial role of tryptase was highlighted by the reduced cell migration observed in HaCaT cells after the treatment with a tryptase inhibitor. To determine the mast cell soluble factors that affect wound healing, we examined the growth factor and cytokine expression in CM from HMC-1 cells and FSL-1 stimulated HMC-1 cells by using a cytokine array. We demonstrated that with or without FSL-1 stimulation, both CM likely expressed IGFBP-2, SCF-R, PDGF-AA, and VEGF-A; RT-PCR also revealed that IGFBP-2 and VEGF-A levels increased in FSL-1-treated HMC-1 cells (Fig 4). The biological functions of the secreted protein, IGFBP-2, include integrin receptor binding (a5b1 and aVb3), extracellular matrix binding, and intracellular interaction; therefore, tumorigenesis and angiogenesis genes, such as *VEGF*, can be expressed [19]. Through proteolytic cleavage, SCF-R (also known as c-Kit or CD117), a tyrosine receptor kinase stem cell factor, can be shed from hematopoietic cells, mast cells, and endothelial cells [20]. PDGF-AA aids in repair by promoting new blood vessel growth [21]. In mouse skin wounds, VEGF-A enhances angiogenesis, wound surface epithelialization, and collagen synthesis [22]. Thus, mast cells participate in wound healing by promoting inflammatory responses through the secretion of various inflammatory mediators. In particular, the mast cell-specific secretion of tryptase, which enhances keratinocyte migration, represents a valuable finding in our study.

In summary, our findings demonstrated that TLR2/6 activation in mast cells promoted early tryptase-dependent wound healing. Therefore, tryptase and other TLR stimulation induced factors that could be considered early wound healing therapeutic candidates.

## Supporting information

S1 Raw data Raw data figures and tables. (ZIP)
