# Supplementary figures and images for: Toll-like receptor 2/6-stimulated HMC-1 mast cells promote keratinocyte migration in wound healing
Source: PLoS One. 2025 Jan 17;20(1):e0317766. doi: 10.1371/journal.pone.0317766 (PMC11741577; doi:10.1371/journal.pone.0317766)

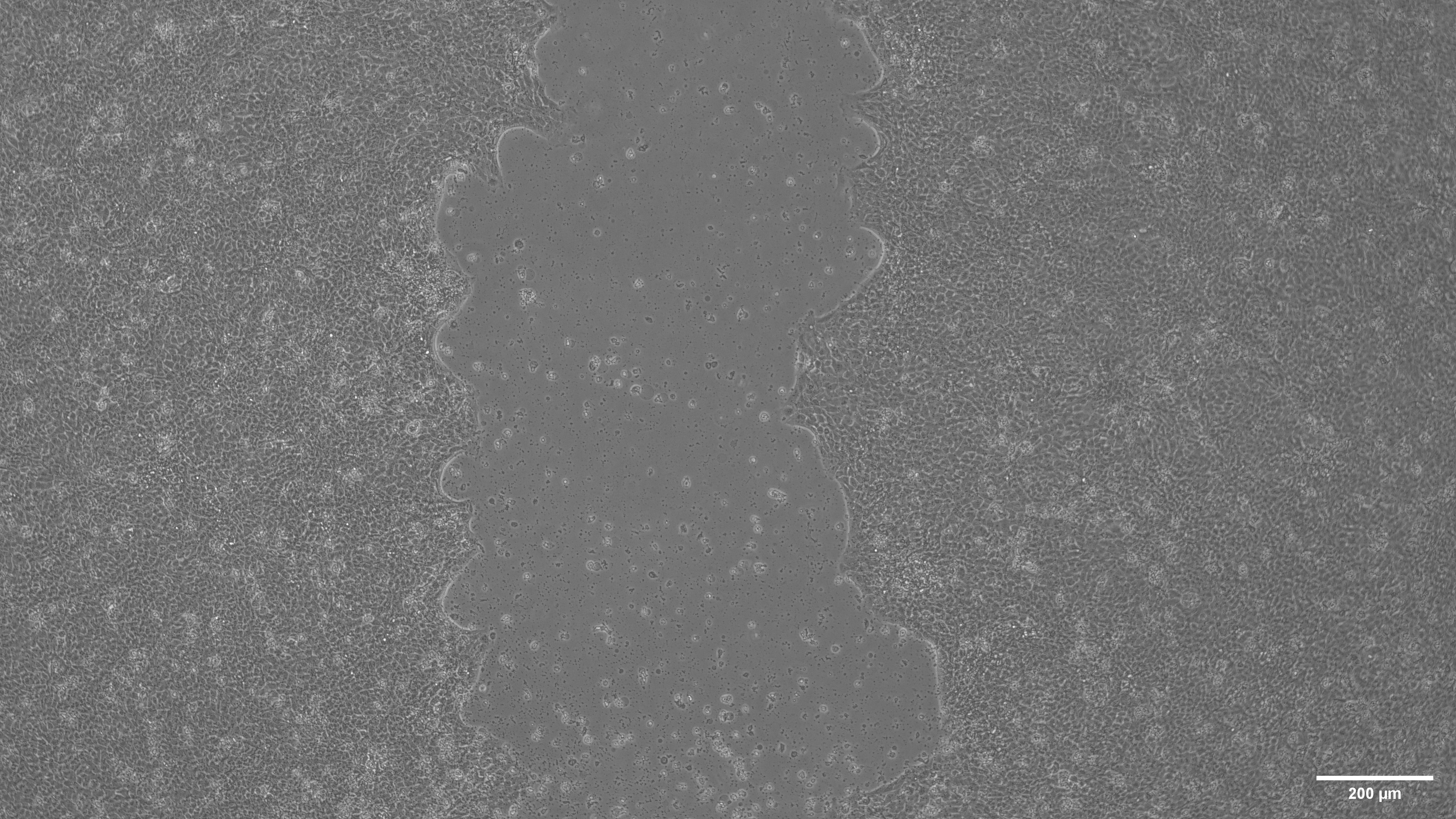

Supplement: S1 Raw data — (ZIP) [file pone.0317766.s001.zip › Archive/5B-C48.jpg]

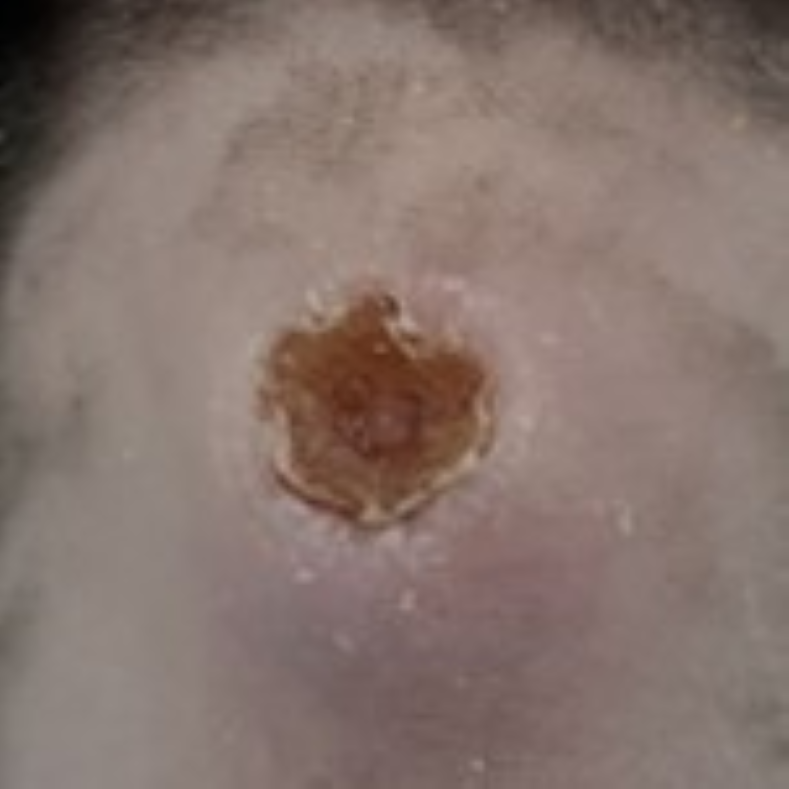

Supplement: S1 Raw data — (ZIP) [file pone.0317766.s001.zip › Archive/3B-day6 FSL-1-HMC-1 CM 300dpi.tif]

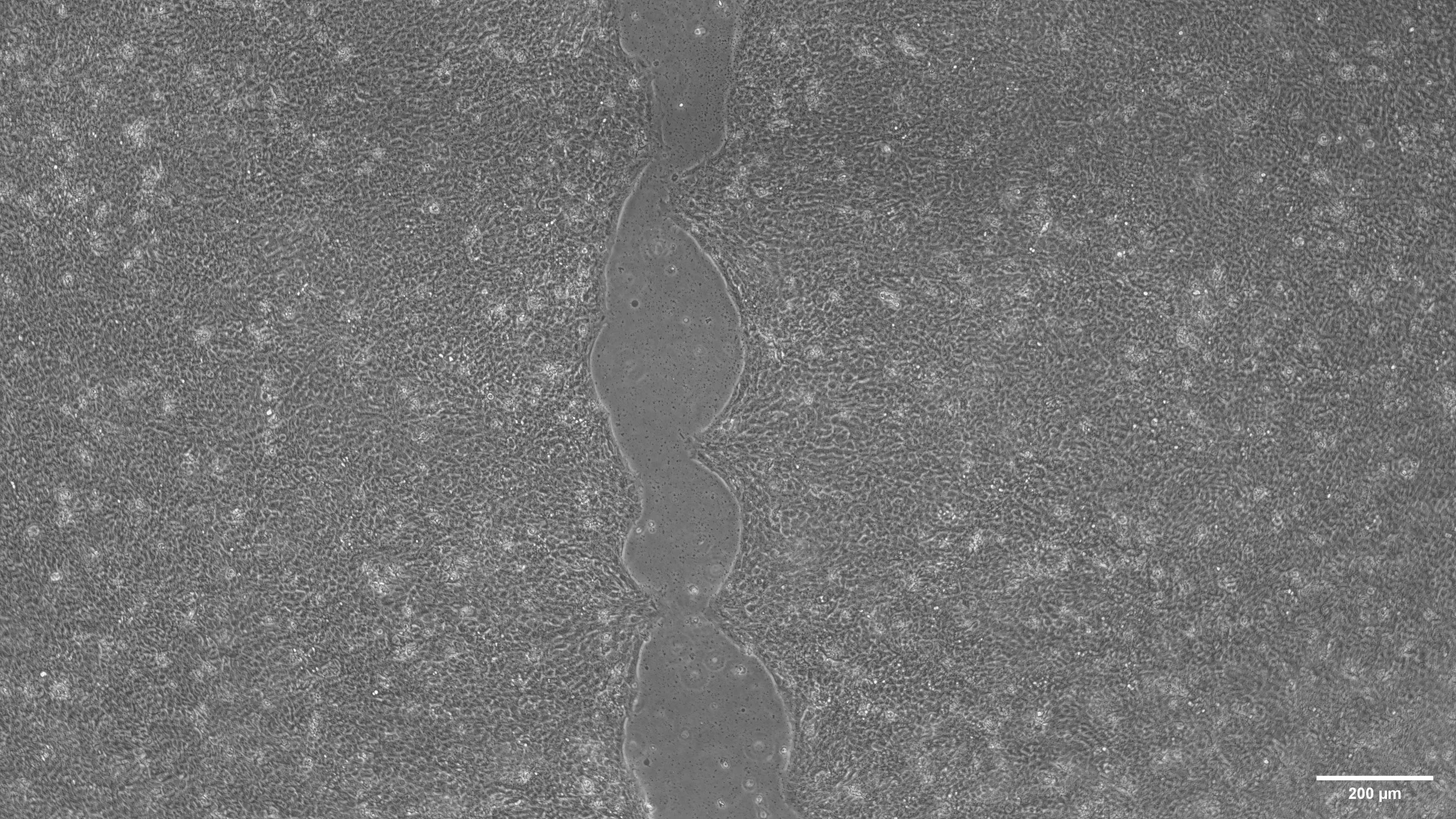

Supplement: S1 Raw data — (ZIP) [file pone.0317766.s001.zip › Archive/5B-FHA48.jpg]

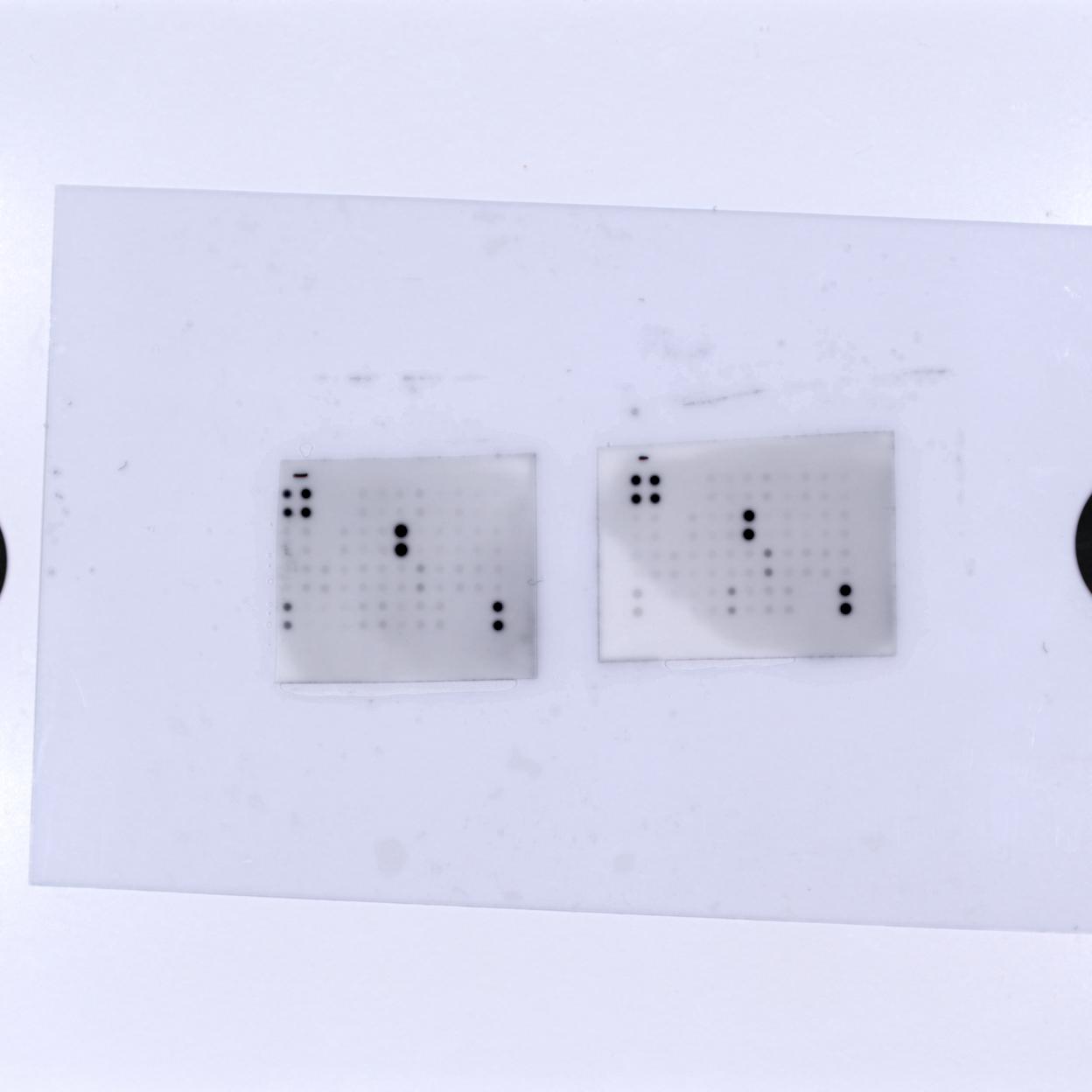

Supplement: S1 Raw data — (ZIP) [file pone.0317766.s001.zip › Archive/4B-20221205-6 20221205_215101_Ch+Marker.jpg]

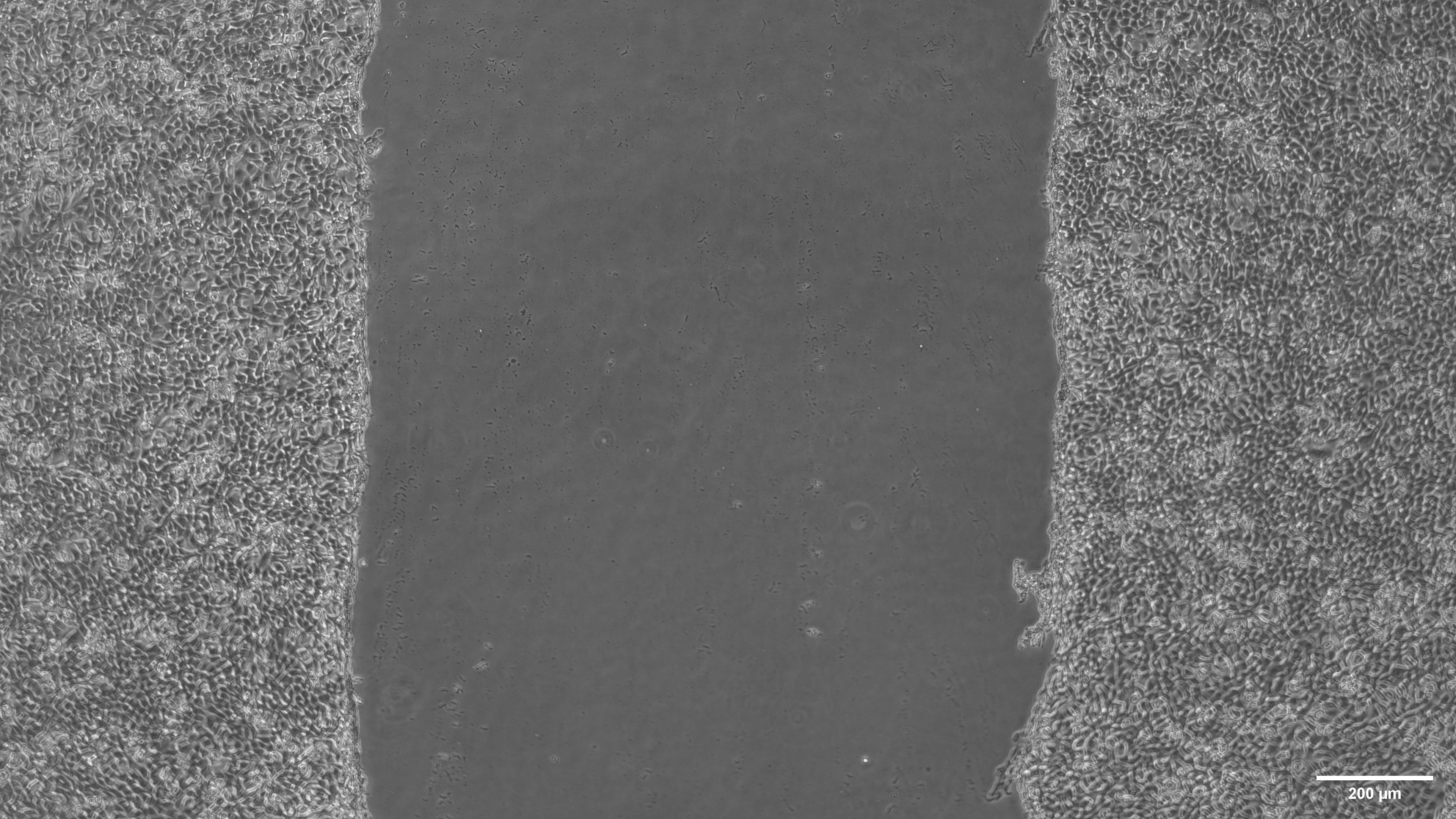

Supplement: S1 Raw data — (ZIP) [file pone.0317766.s001.zip › Archive/5B-CA0.jpg]

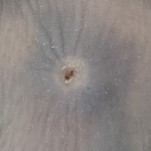

Supplement: S1 Raw data — (ZIP) [file pone.0317766.s001.zip › Archive/3B-day12 tlr2and6300.jpg]

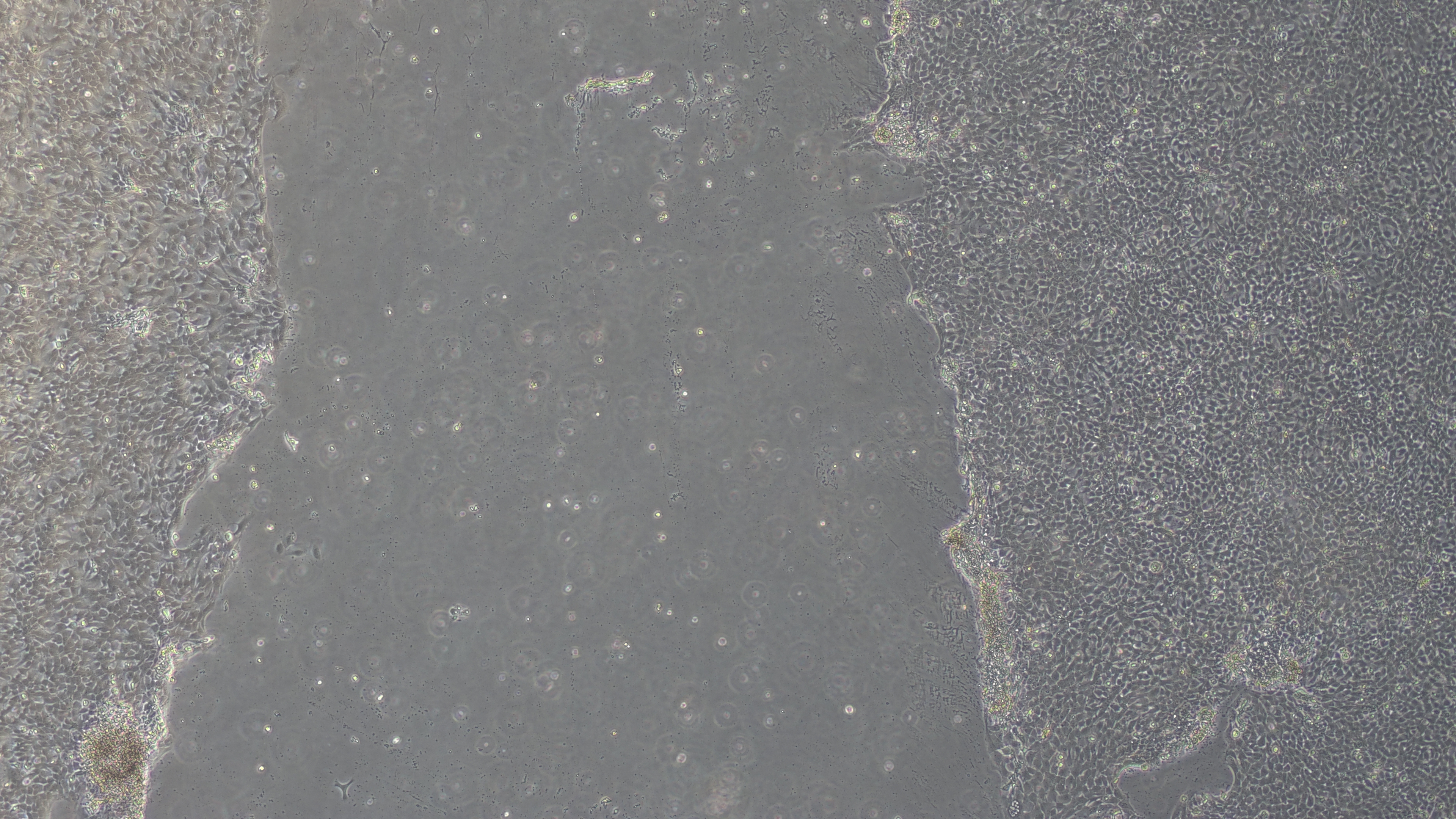

Supplement: S1 Raw data — (ZIP) [file pone.0317766.s001.zip › Archive/1A-C24 h.jpg]

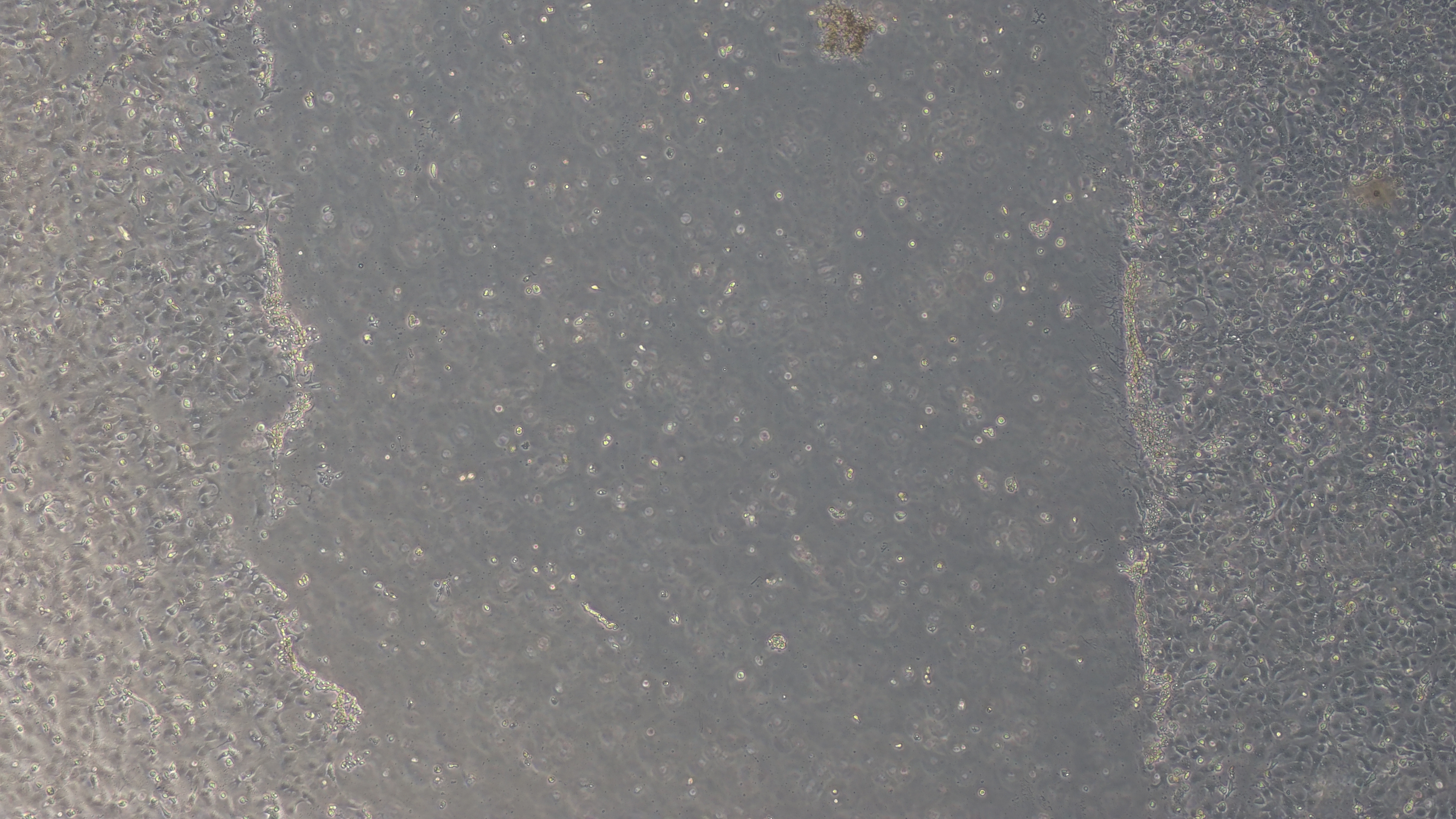

Supplement: S1 Raw data — (ZIP) [file pone.0317766.s001.zip › Archive/1A-mito48 h.jpg]

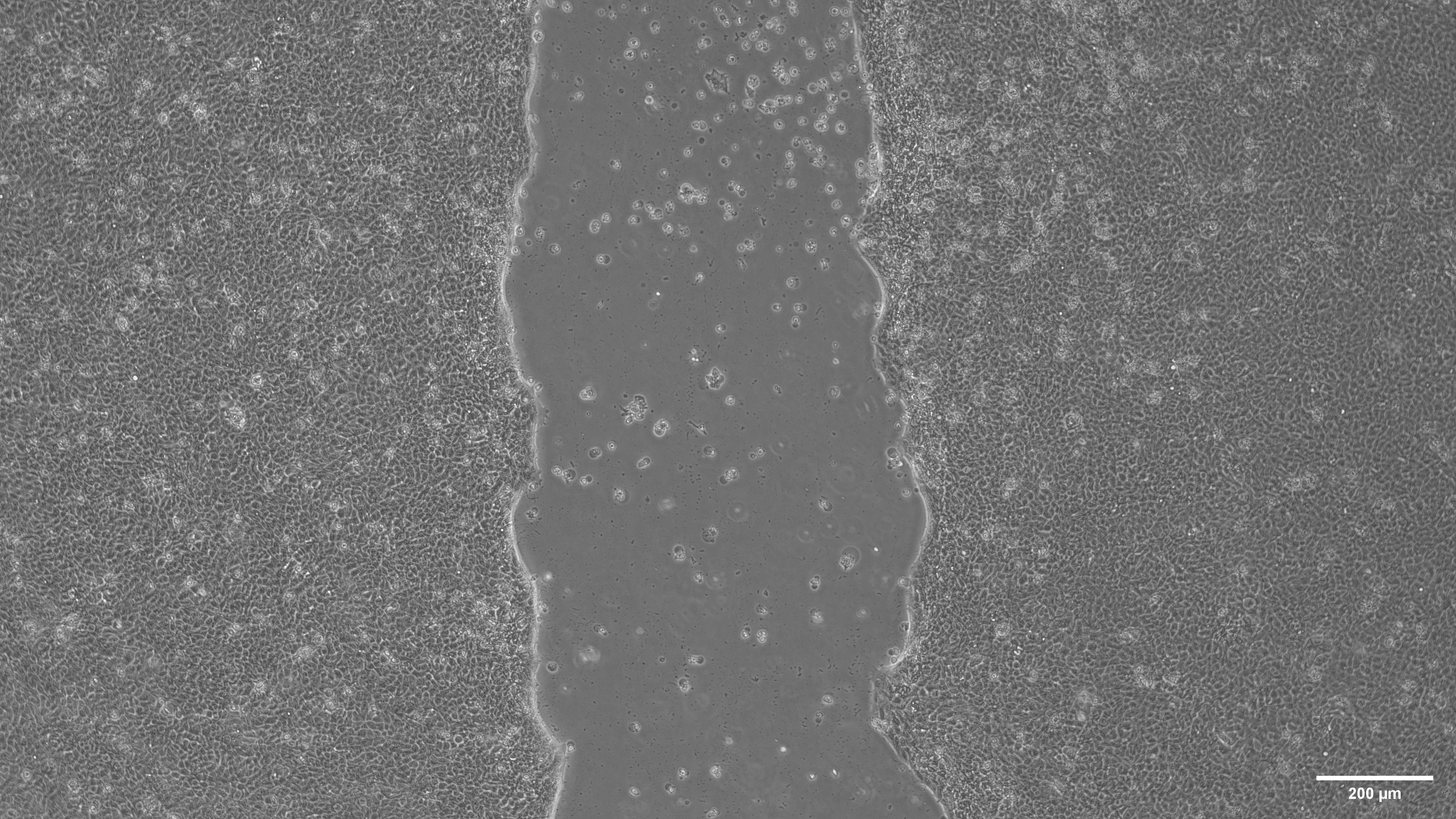

Supplement: S1 Raw data — (ZIP) [file pone.0317766.s001.zip › Archive/5B-H24.jpg]

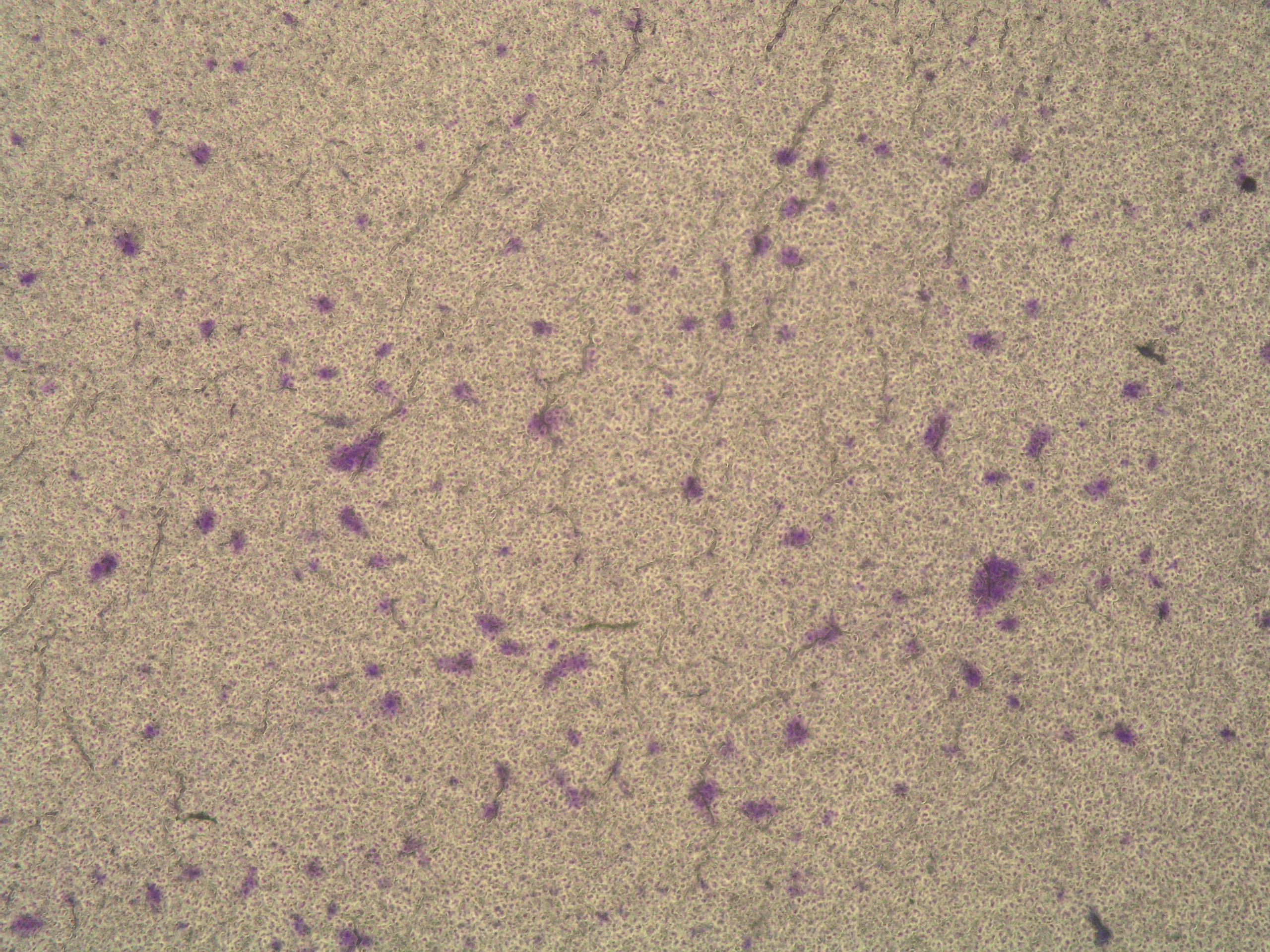

Supplement: S1 Raw data — (ZIP) [file pone.0317766.s001.zip › Archive/1C-HMC-1 CM 0h (300dpi).JPG]

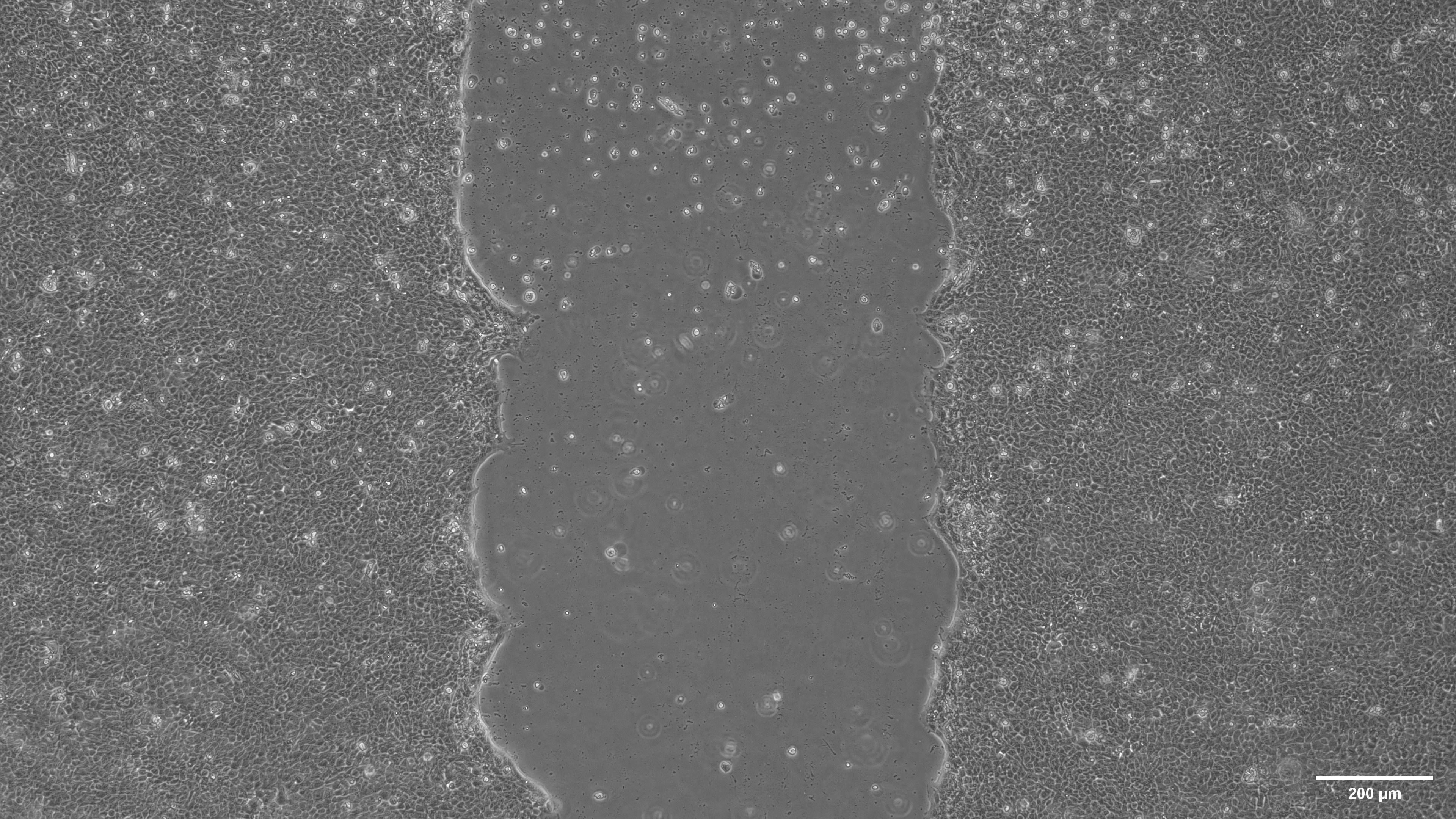

Supplement: S1 Raw data — (ZIP) [file pone.0317766.s001.zip › Archive/5B-CA24.jpg]

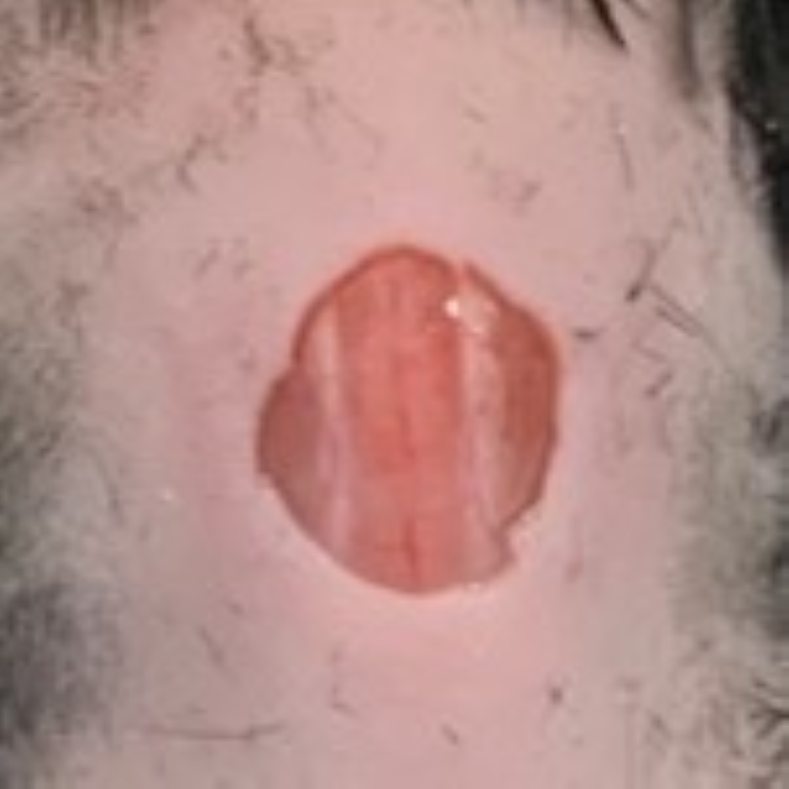

Supplement: S1 Raw data — (ZIP) [file pone.0317766.s001.zip › Archive/3B-day1 FSL-1-HMC-1 CM 300dpi.tif]

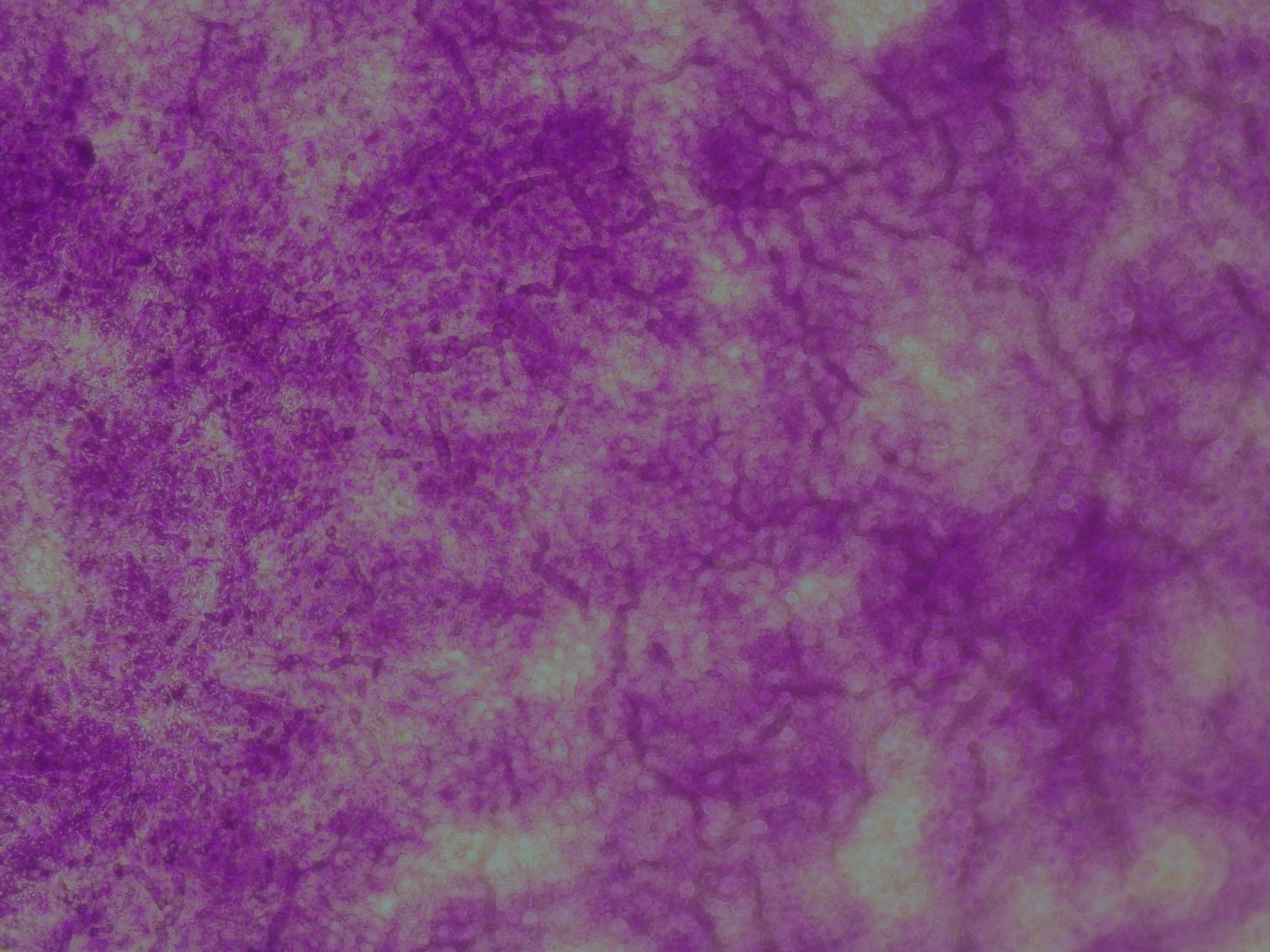

Supplement: S1 Raw data — (ZIP) [file pone.0317766.s001.zip › Archive/1C-HMC-1 CM 24h (300dpi).JPG]

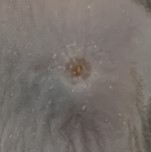

Supplement: S1 Raw data — (ZIP) [file pone.0317766.s001.zip › Archive/3B-day12 control300.jpg]

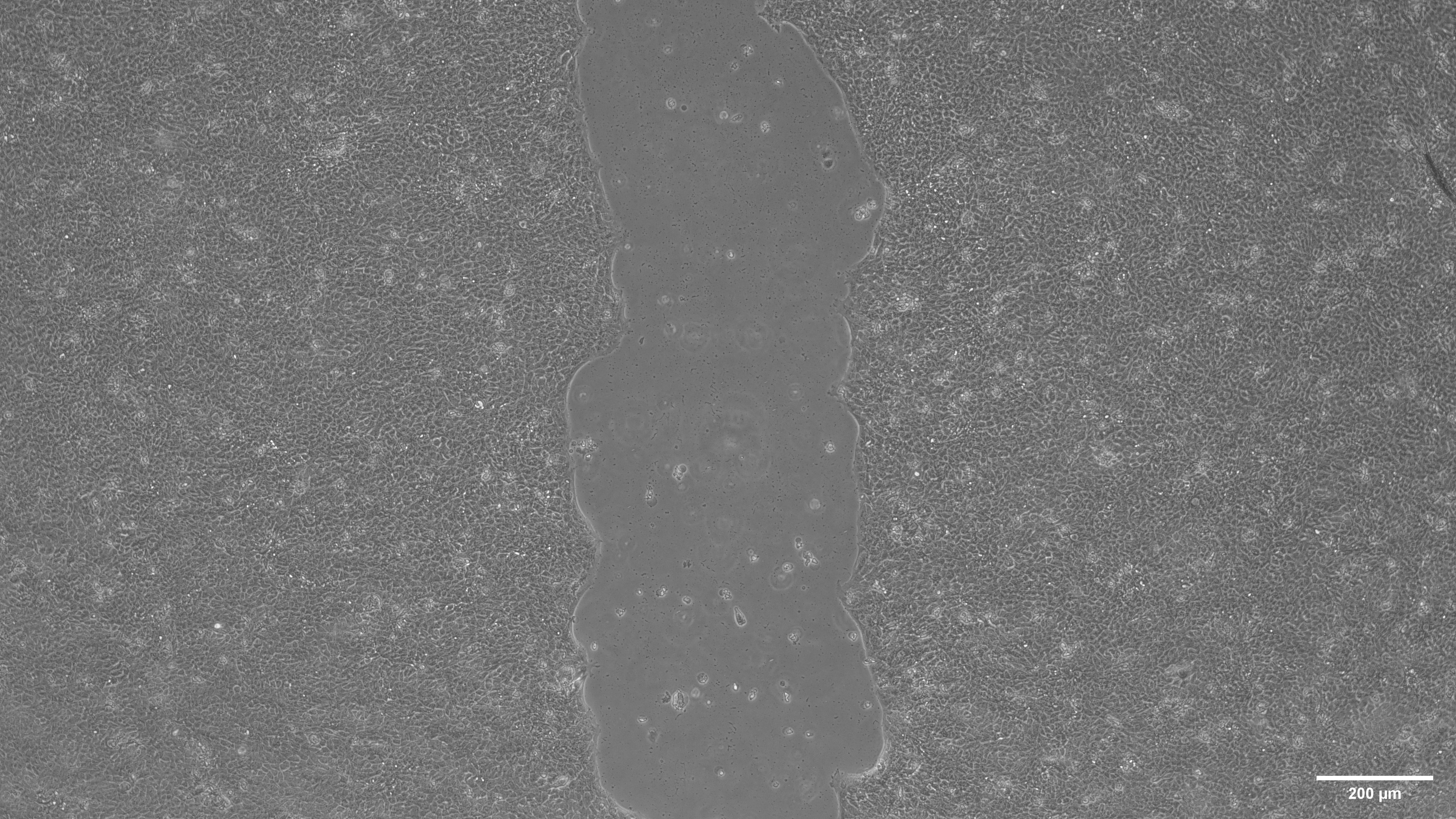

Supplement: S1 Raw data — (ZIP) [file pone.0317766.s001.zip › Archive/5B-HA48.jpg]

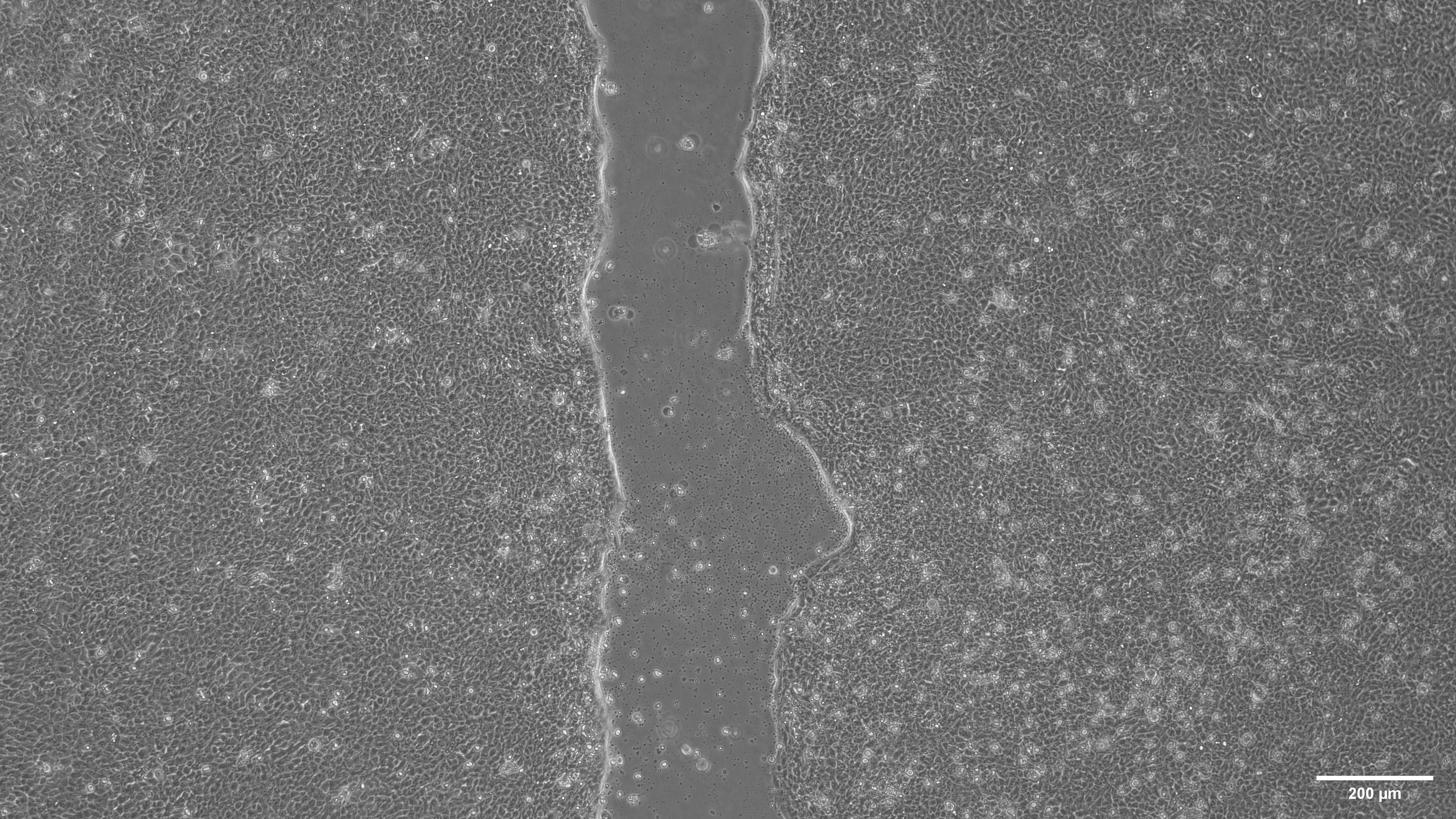

Supplement: S1 Raw data — (ZIP) [file pone.0317766.s001.zip › Archive/5B-FH24.jpg]

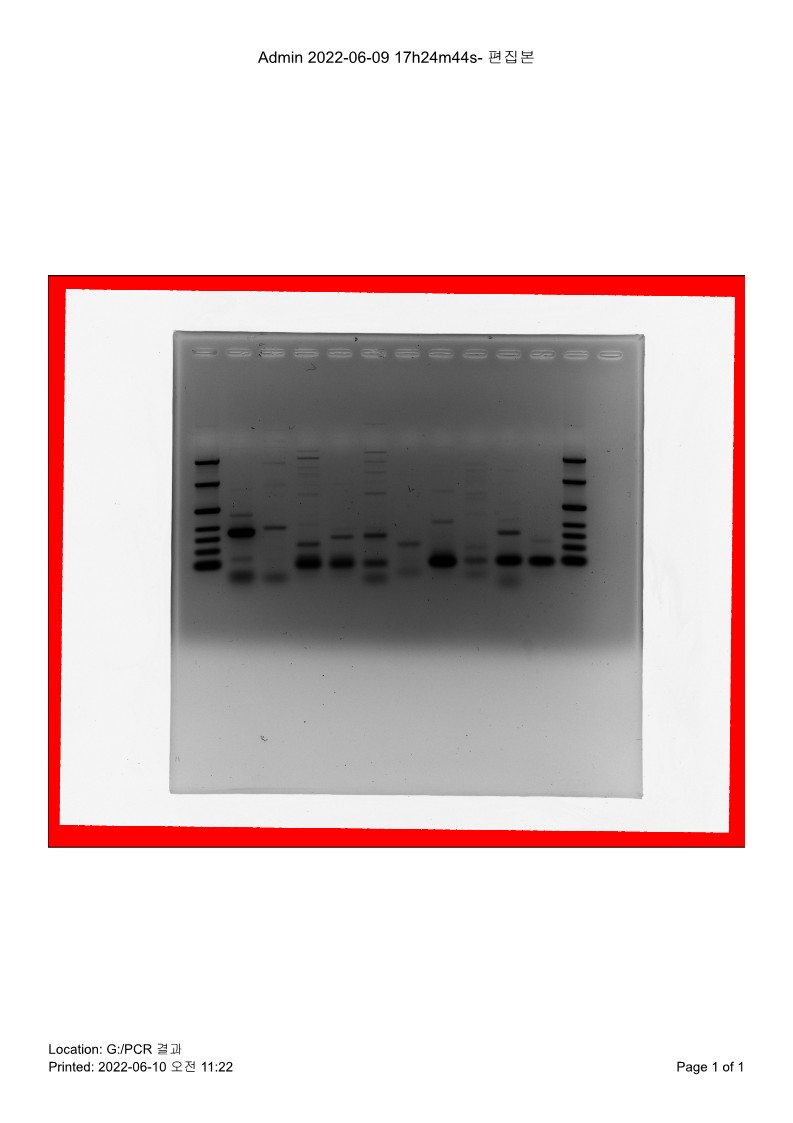

Supplement: S1 Raw data — (ZIP) [file pone.0317766.s001.zip › Archive/2B-(orignal)Admin 2022-06-09 17h24m44s.jpg]

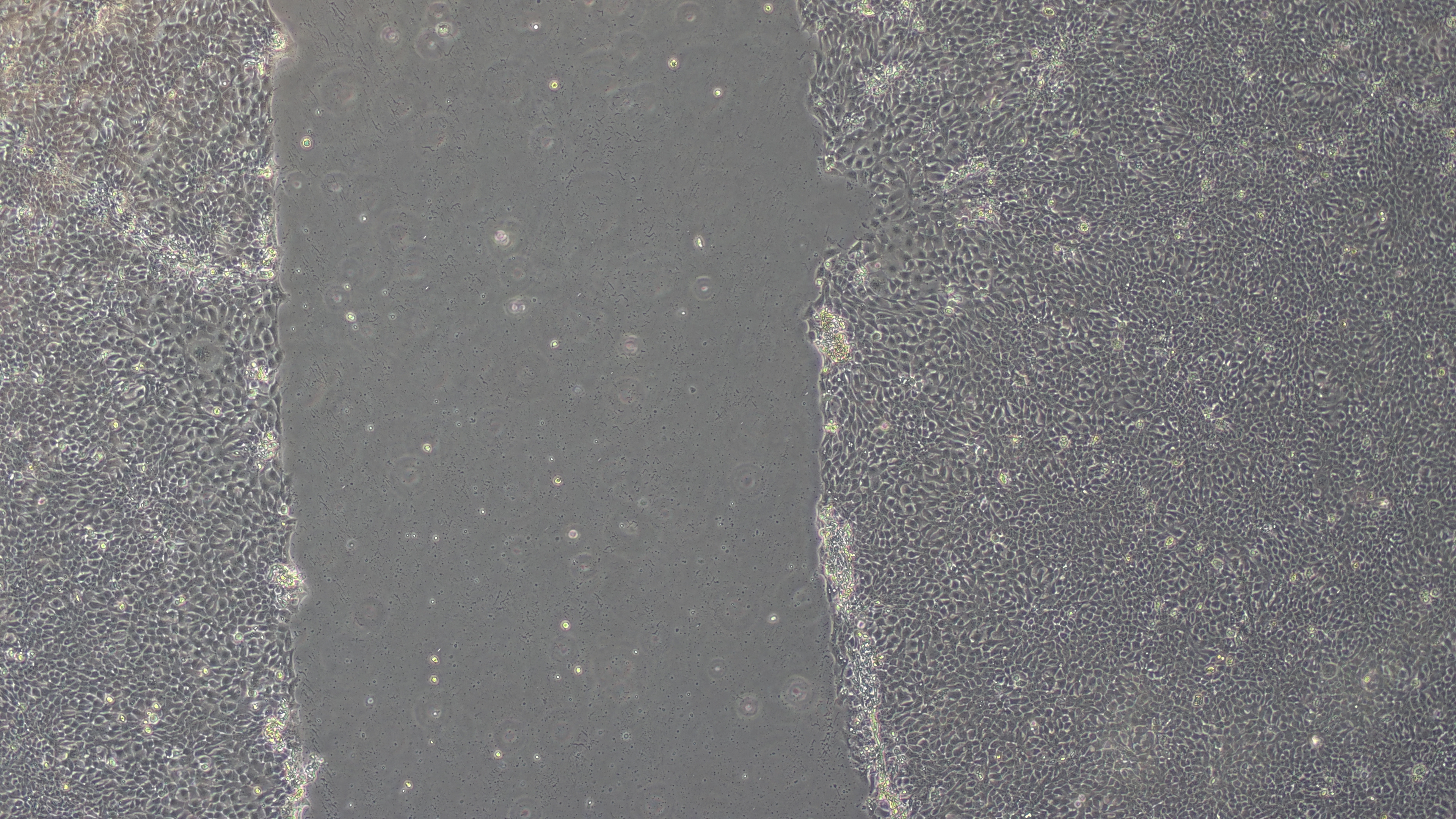

Supplement: S1 Raw data — (ZIP) [file pone.0317766.s001.zip › Archive/1A-H24 h.jpg]

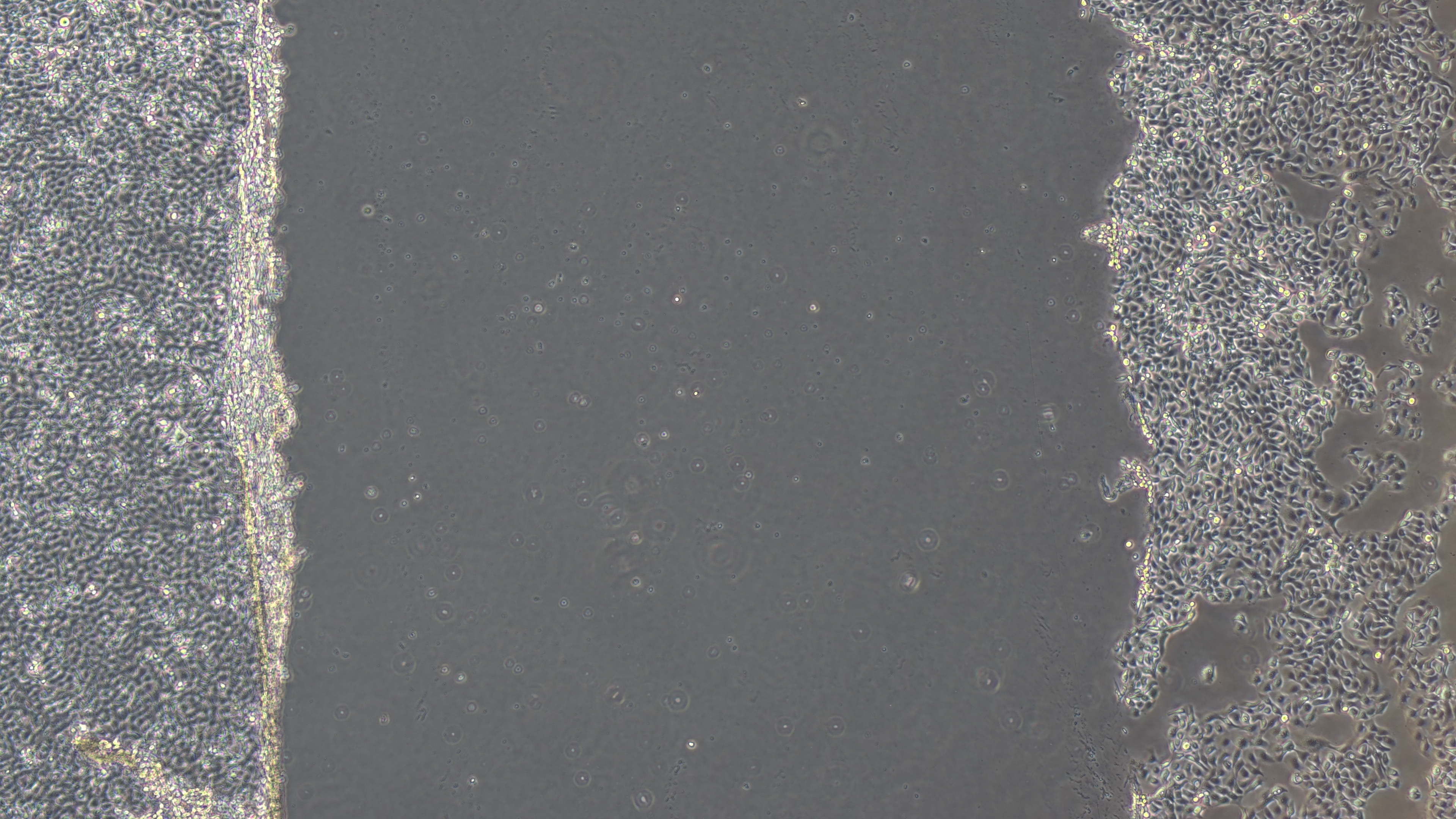

Supplement: S1 Raw data — (ZIP) [file pone.0317766.s001.zip › Archive/1A-C0 h.jpg]

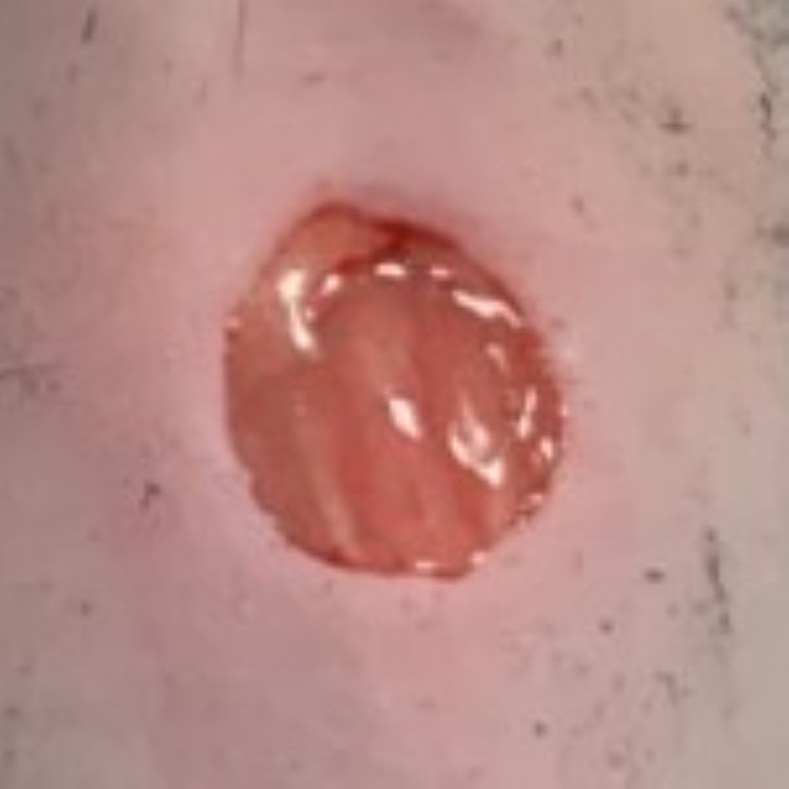

Supplement: S1 Raw data — (ZIP) [file pone.0317766.s001.zip › Archive/3B-day1 FSL-1 300dpi.tif]

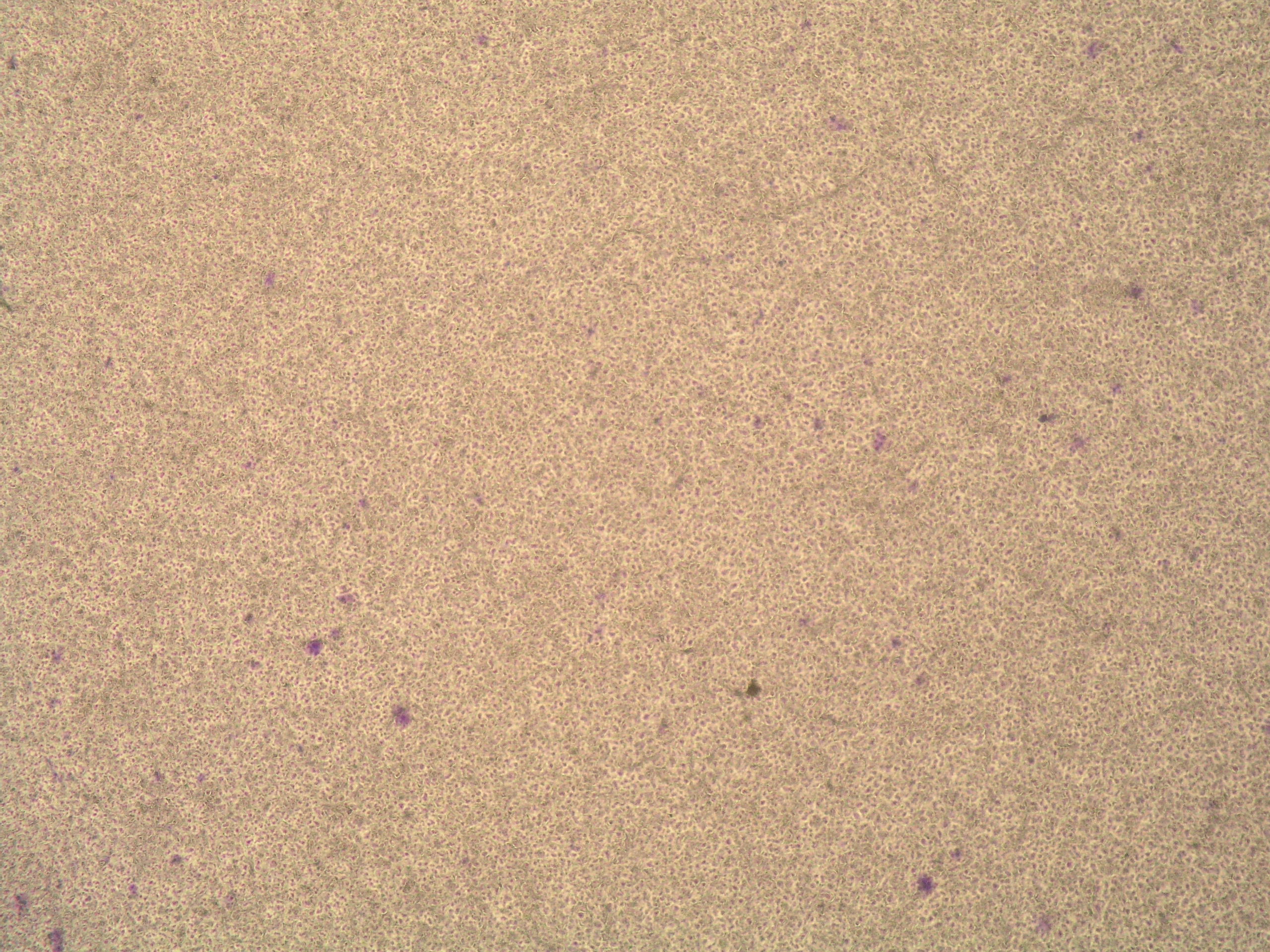

Supplement: S1 Raw data — (ZIP) [file pone.0317766.s001.zip › Archive/1C-Control medium 0h (300dpi).JPG]

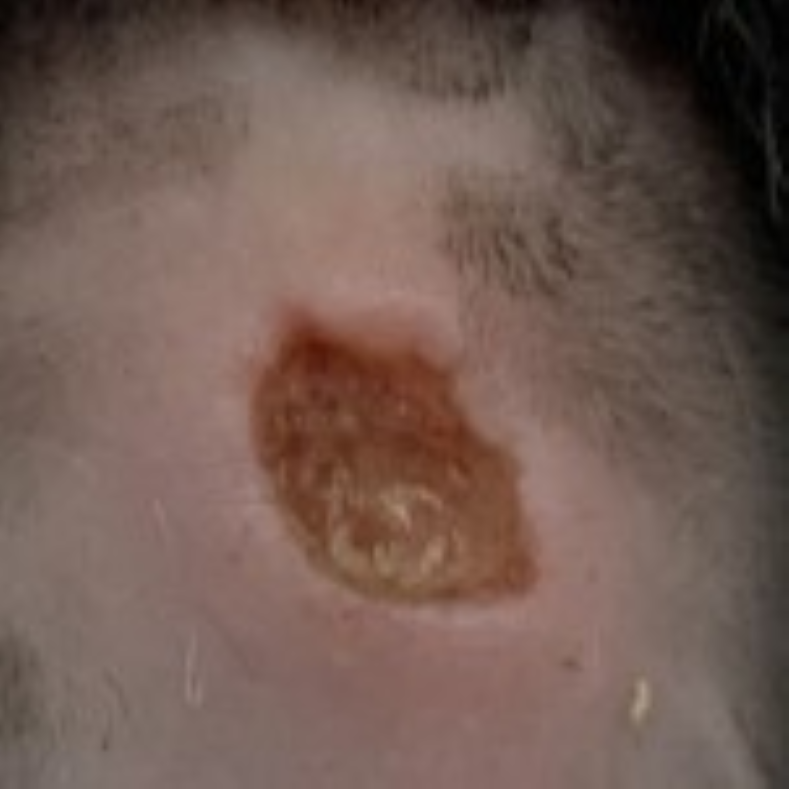

Supplement: S1 Raw data — (ZIP) [file pone.0317766.s001.zip › Archive/3B-day3 FSL-1-HMC-1 CM 300dpi.tif]

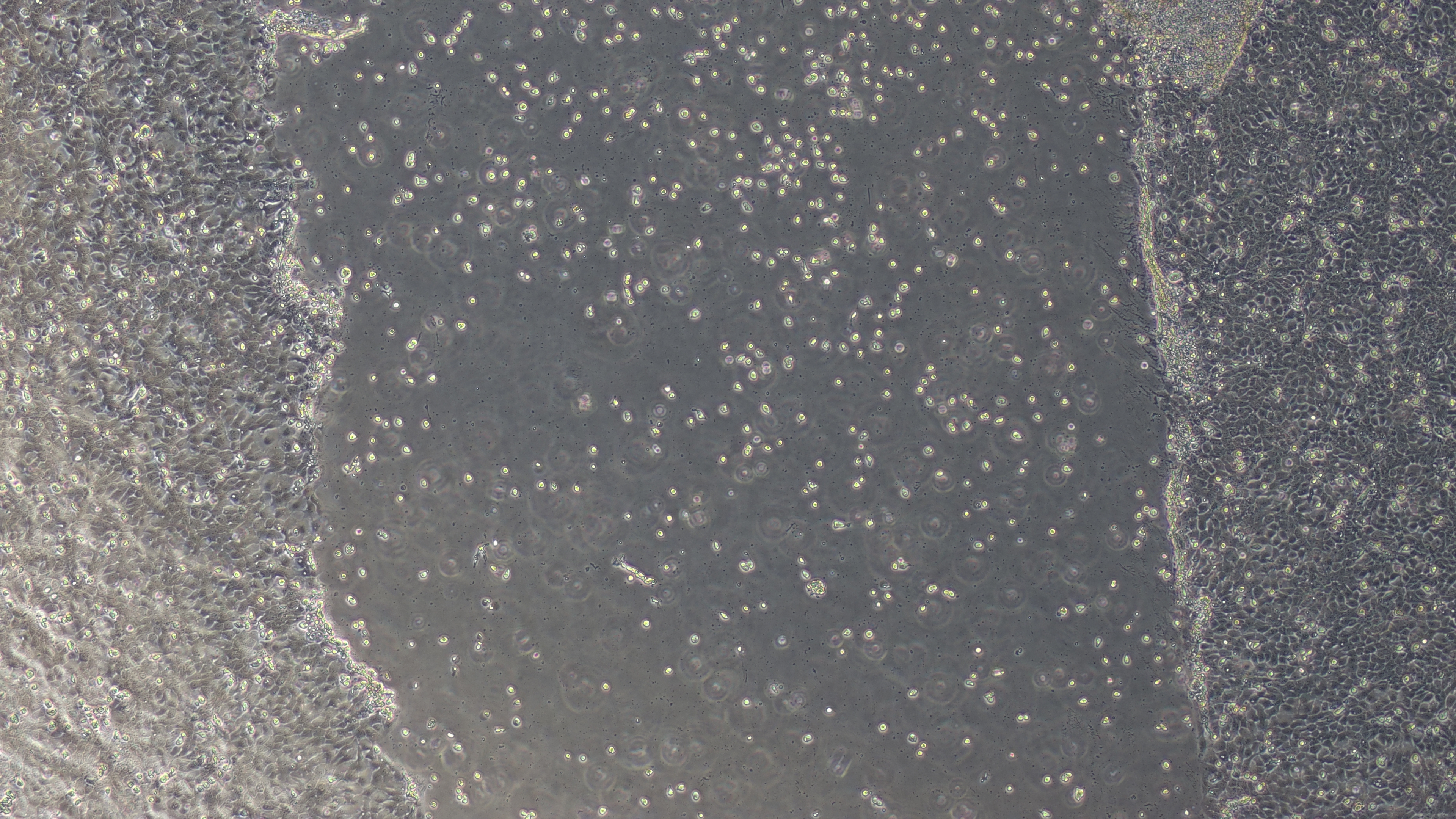

Supplement: S1 Raw data — (ZIP) [file pone.0317766.s001.zip › Archive/1A-mito24 h.jpg]

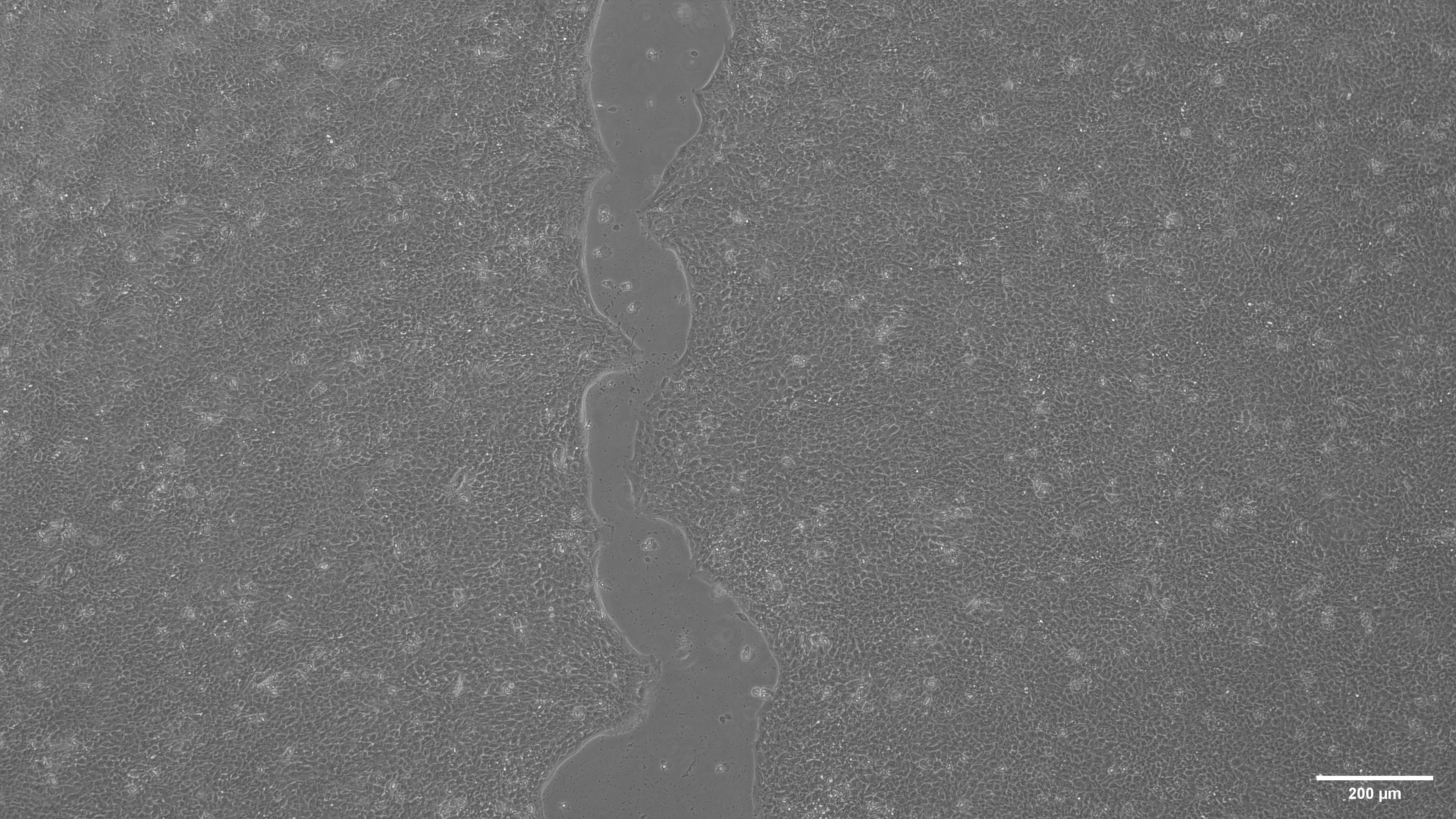

Supplement: S1 Raw data — (ZIP) [file pone.0317766.s001.zip › Archive/5B-H48.jpg]

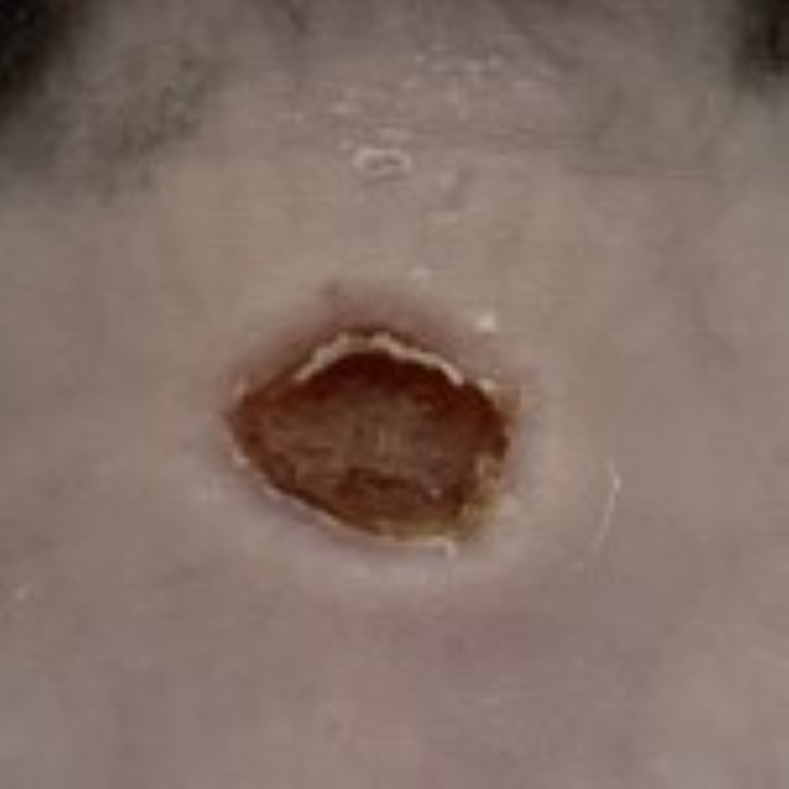

Supplement: S1 Raw data — (ZIP) [file pone.0317766.s001.zip › Archive/3B-day6 HMC-1 CM 300dpi.tif]

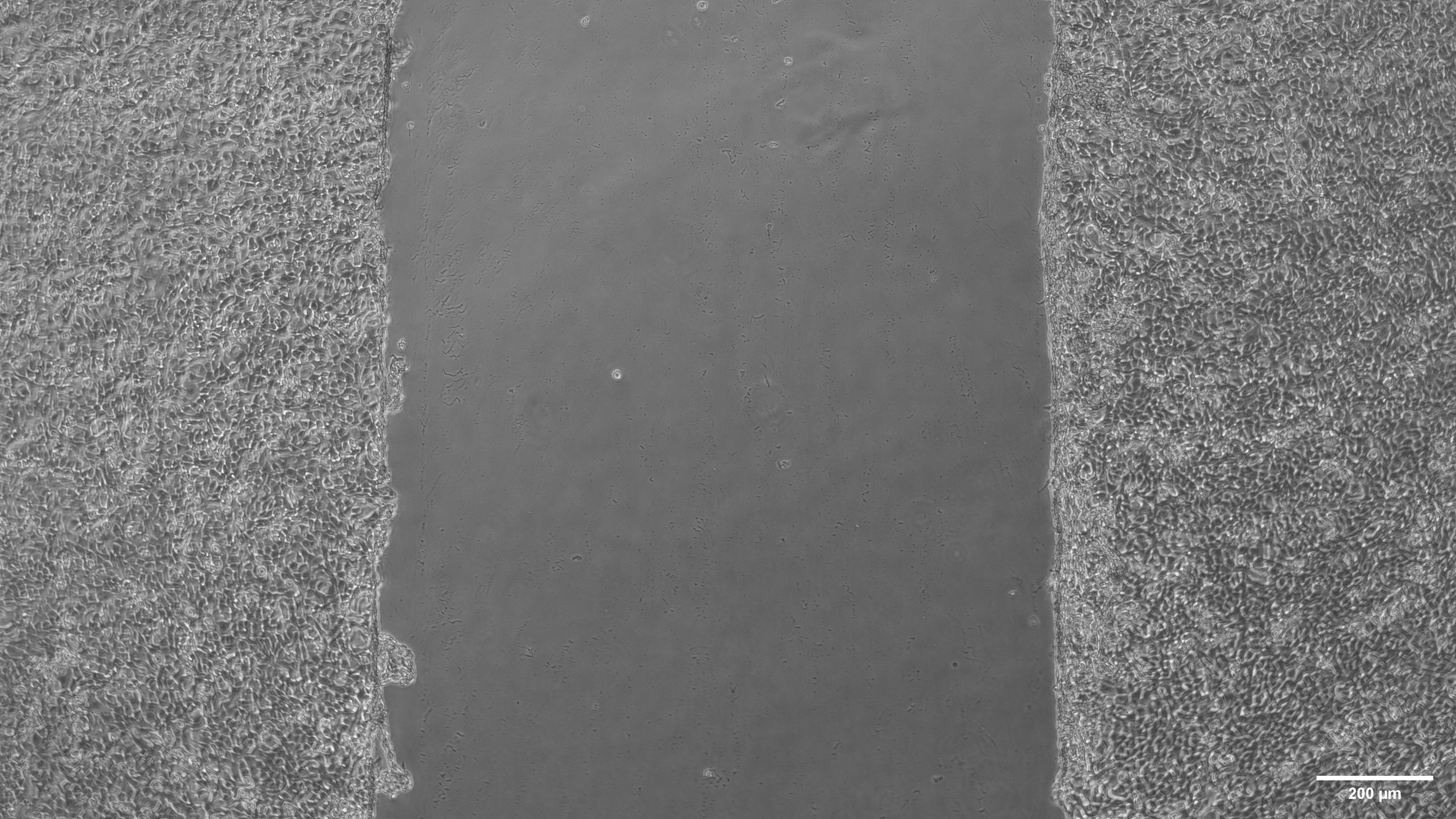

Supplement: S1 Raw data — (ZIP) [file pone.0317766.s001.zip › Archive/5B-H0.jpg]

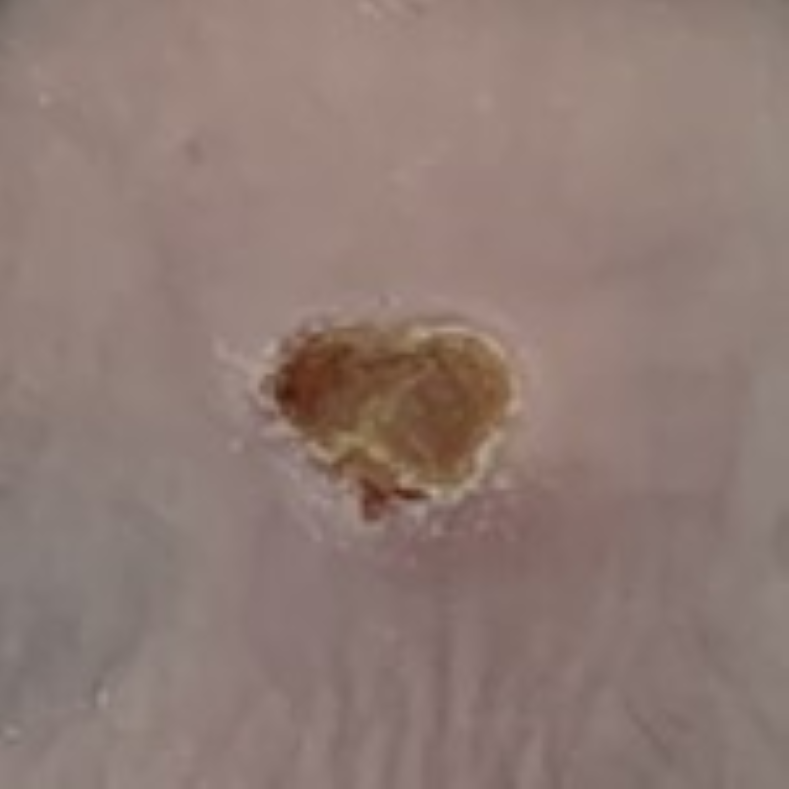

Supplement: S1 Raw data — (ZIP) [file pone.0317766.s001.zip › Archive/3B-day6 FSL-1 300dpi.tif]

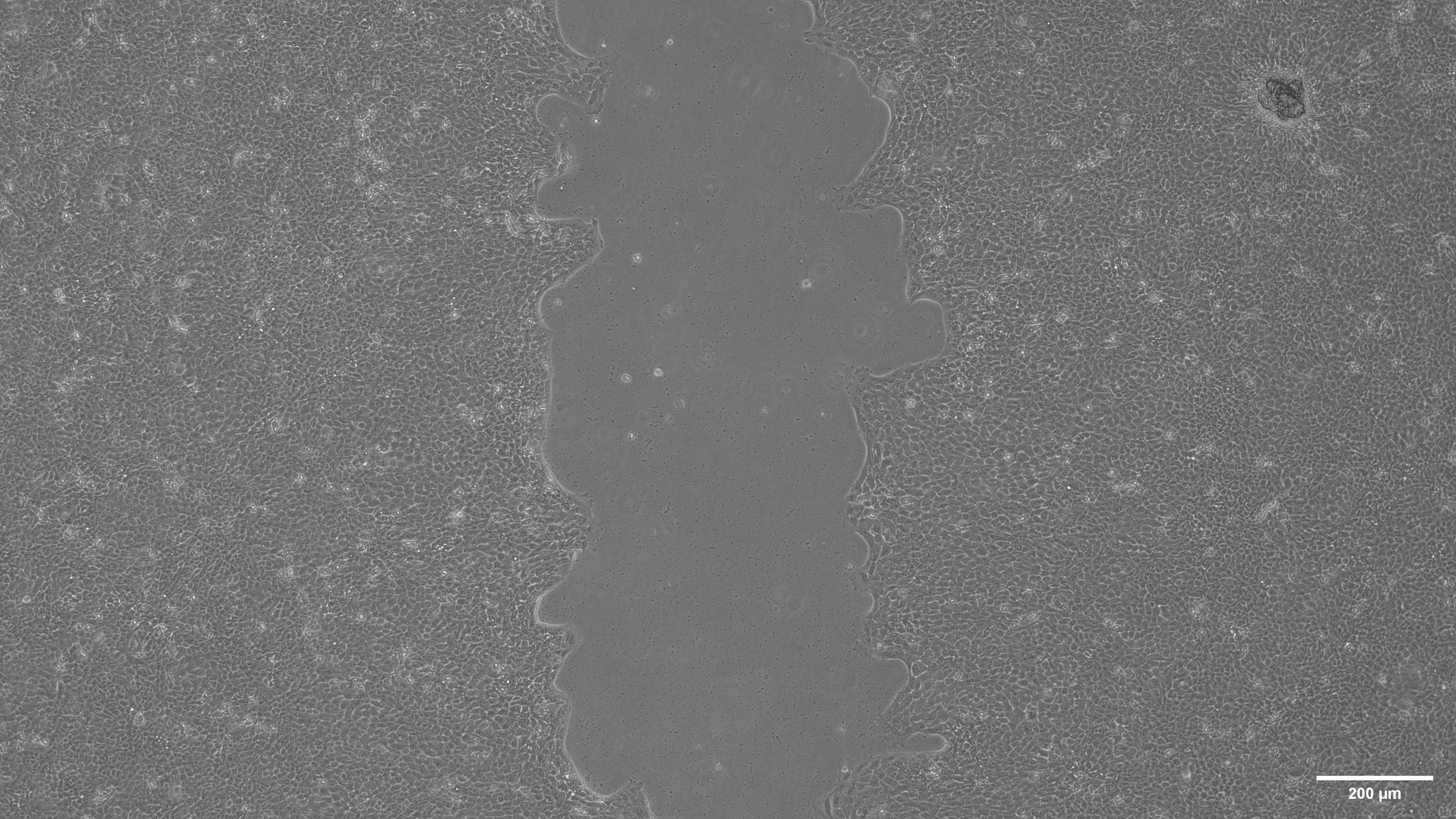

Supplement: S1 Raw data — (ZIP) [file pone.0317766.s001.zip › Archive/5B-CA48.jpg]

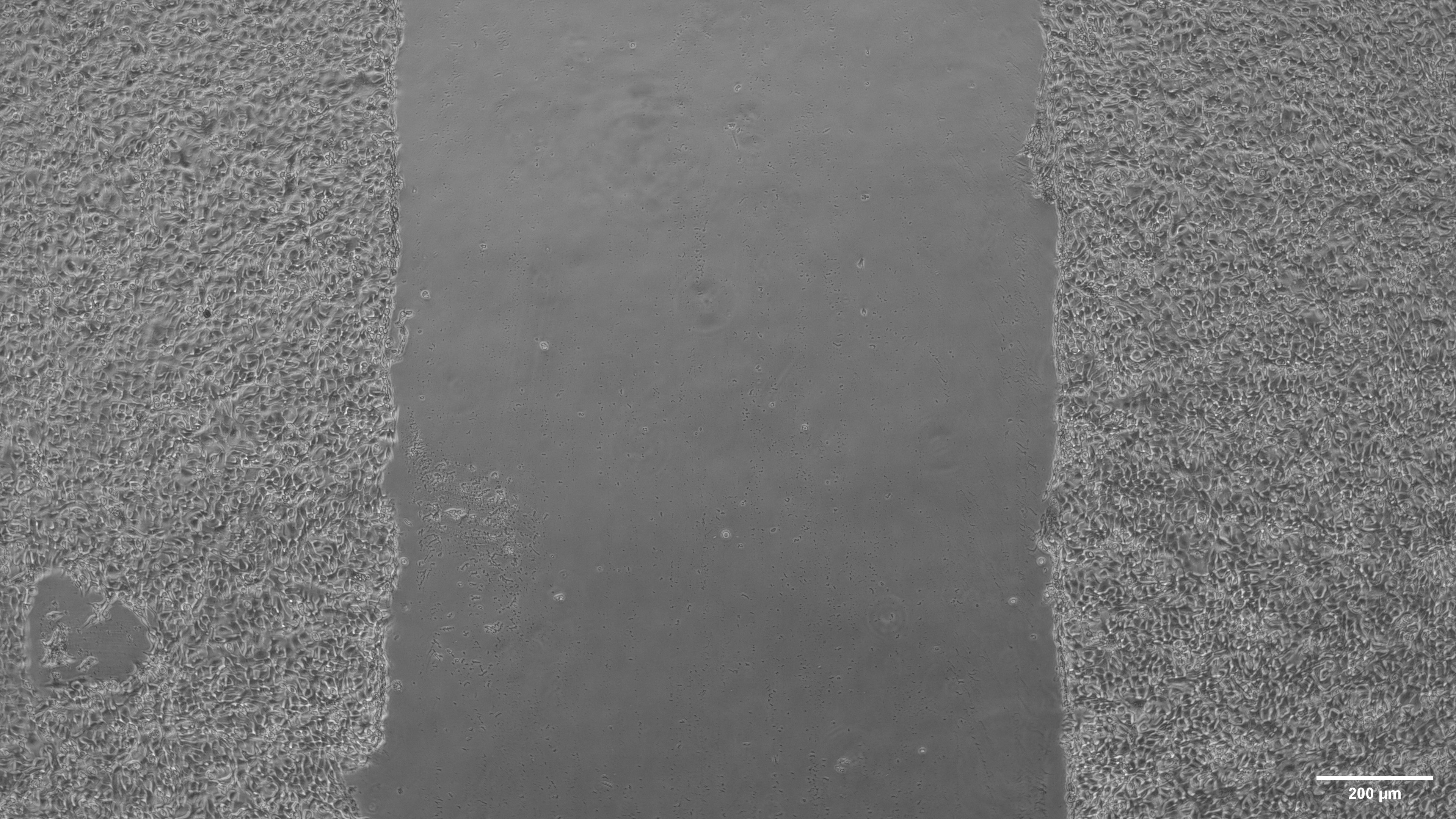

Supplement: S1 Raw data — (ZIP) [file pone.0317766.s001.zip › Archive/5B-HA0.jpg]

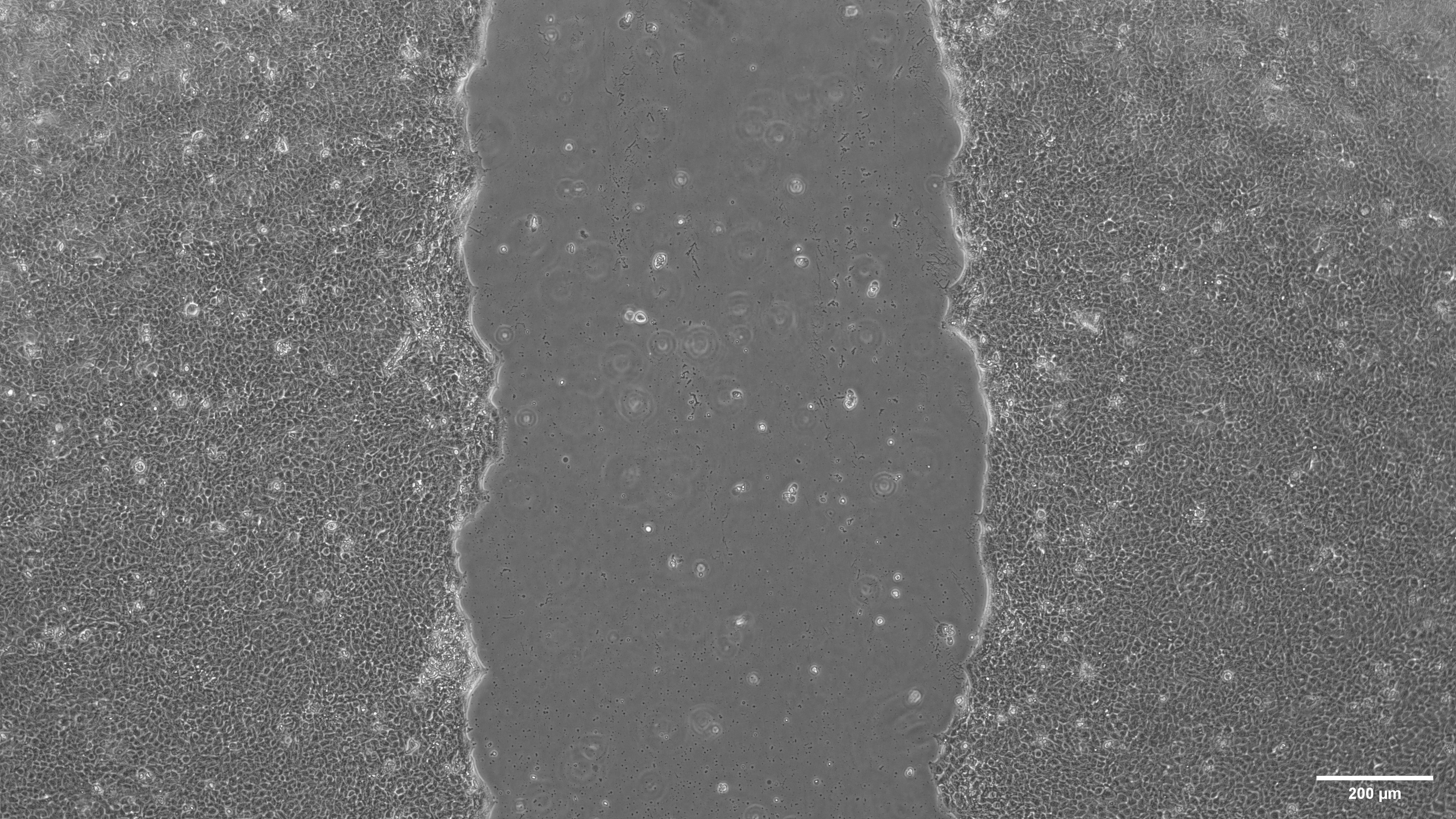

Supplement: S1 Raw data — (ZIP) [file pone.0317766.s001.zip › Archive/5B-C24.jpg]

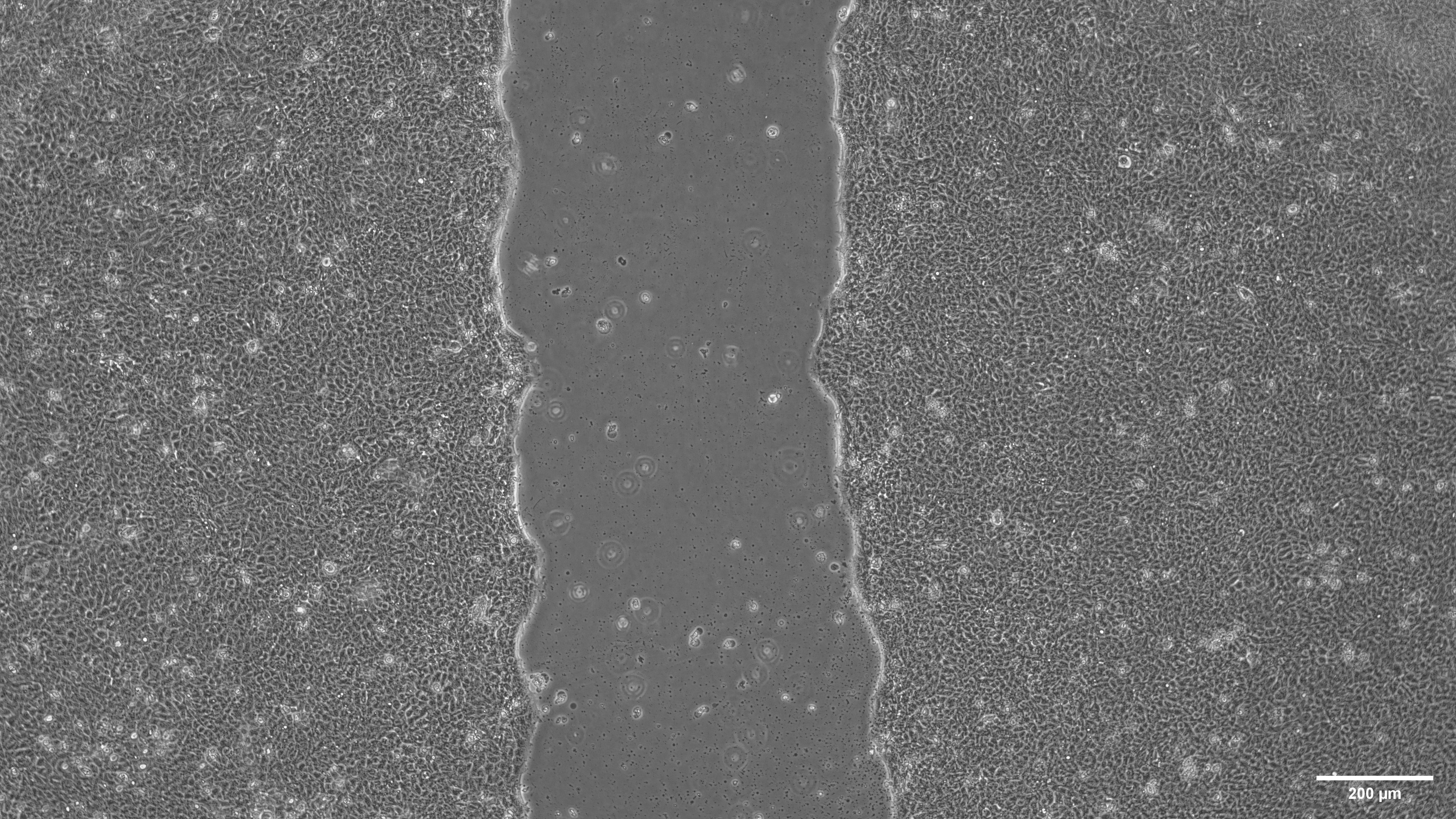

Supplement: S1 Raw data — (ZIP) [file pone.0317766.s001.zip › Archive/5B-FHA24.jpg]

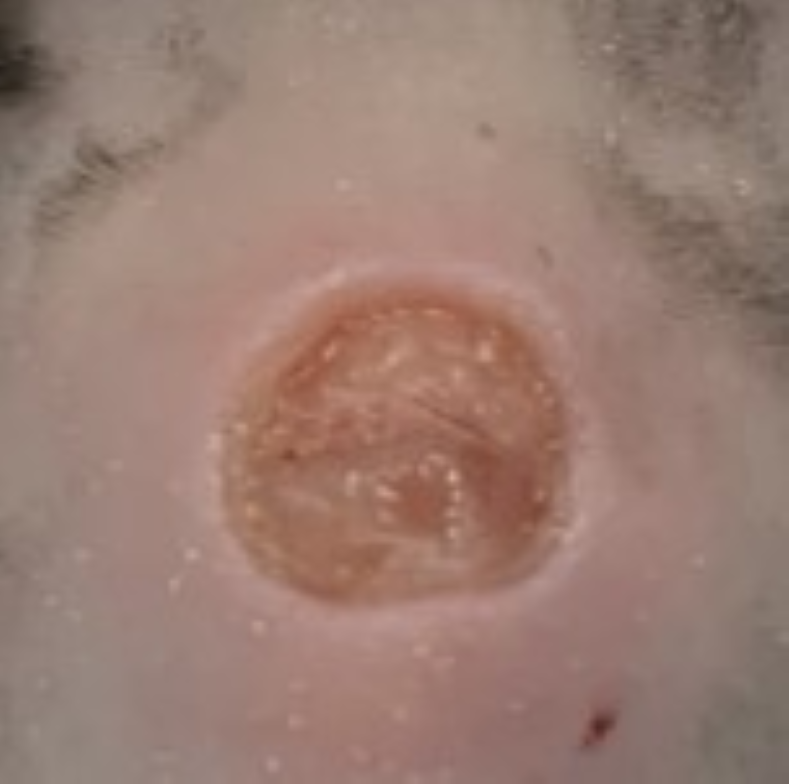

Supplement: S1 Raw data — (ZIP) [file pone.0317766.s001.zip › Archive/3B-day3 HMC-1 CM 300dpi.tif]

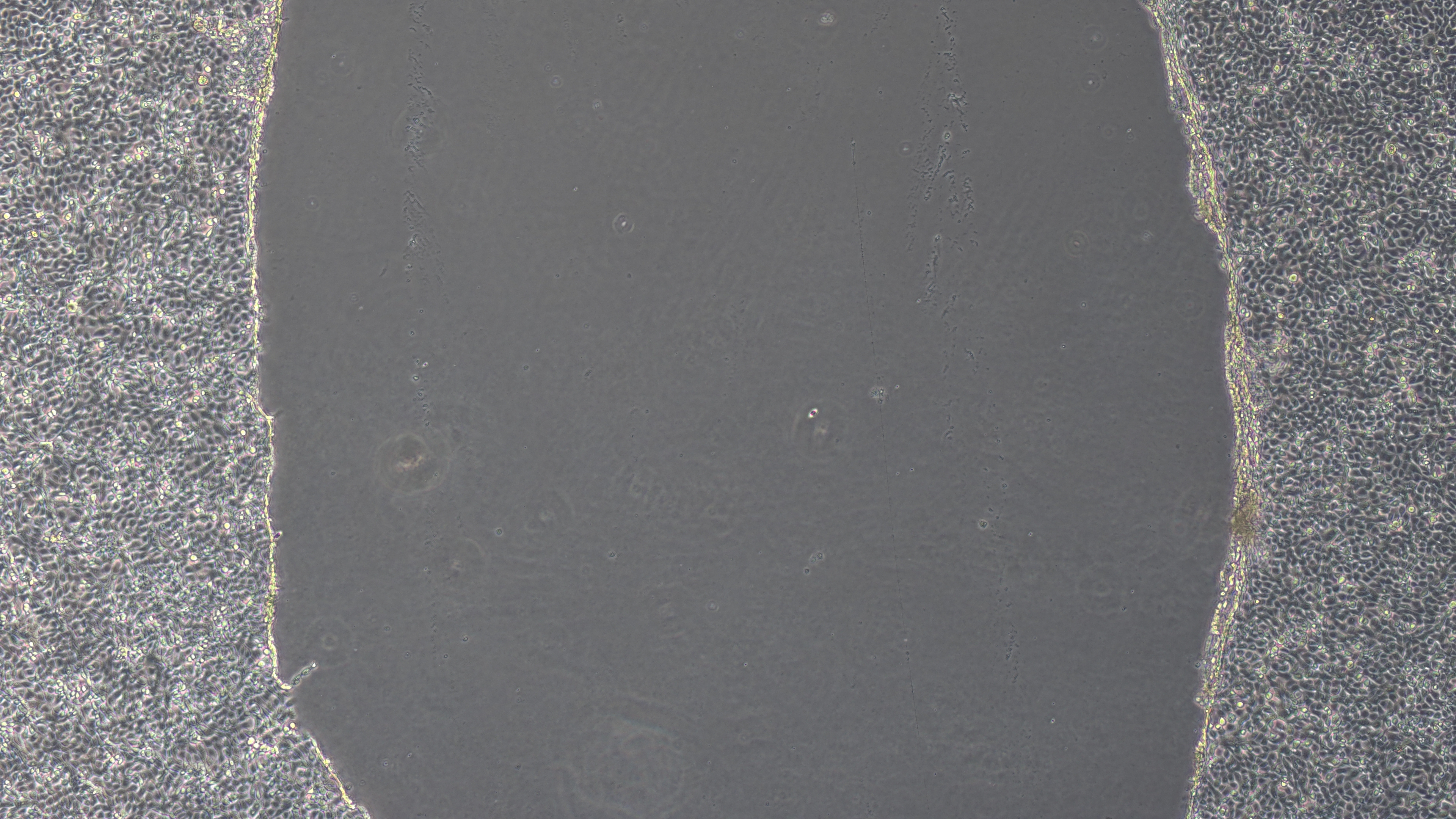

Supplement: S1 Raw data — (ZIP) [file pone.0317766.s001.zip › Archive/1A-H0 h.jpg]

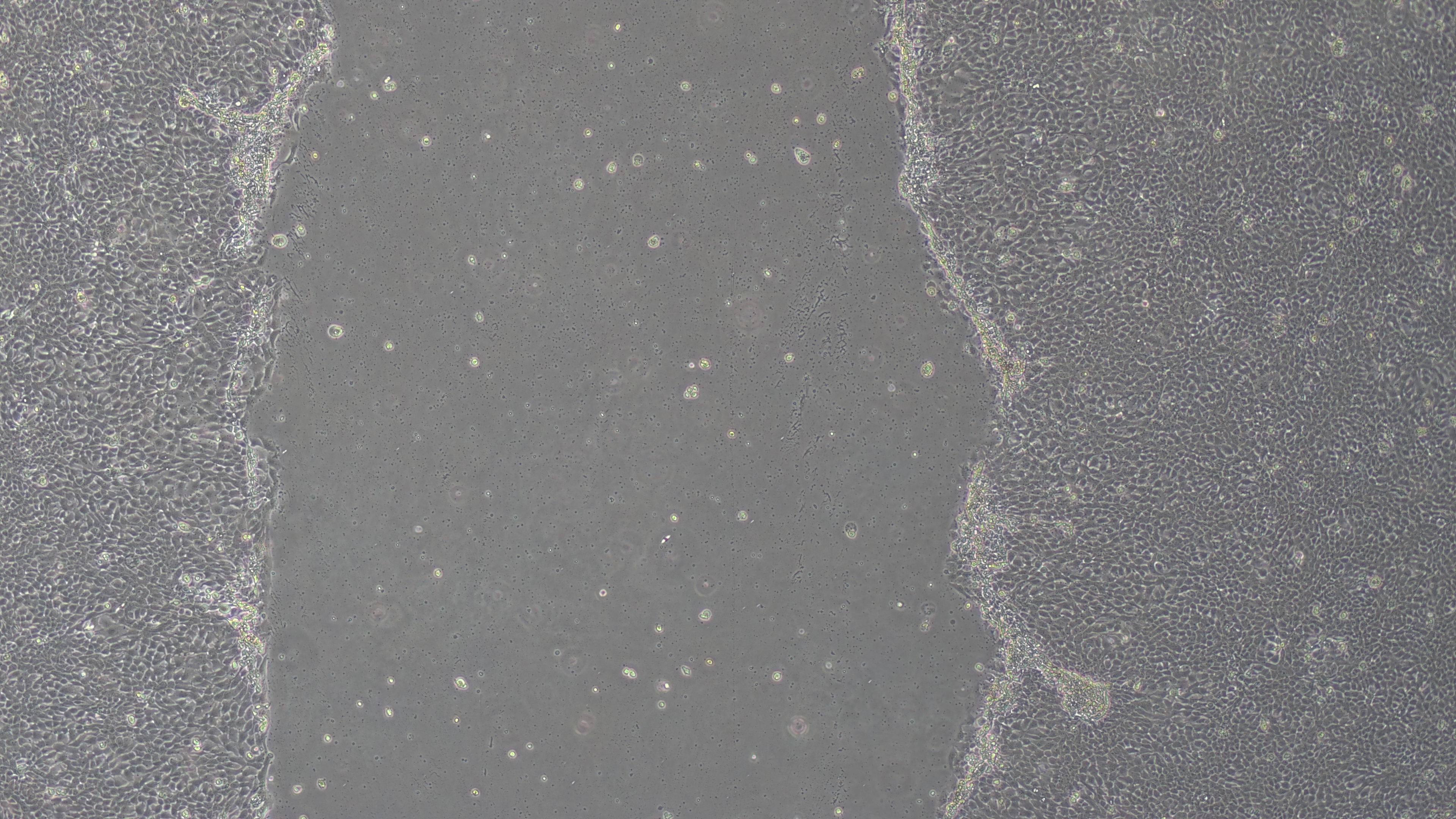

Supplement: S1 Raw data — (ZIP) [file pone.0317766.s001.zip › Archive/1A-C48 h.jpg]

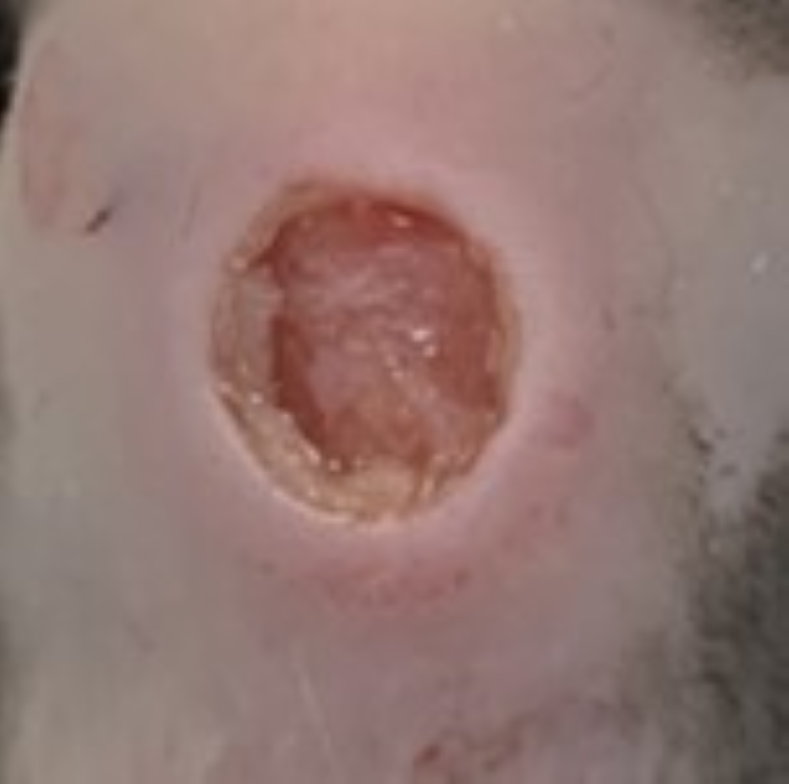

Supplement: S1 Raw data — (ZIP) [file pone.0317766.s001.zip › Archive/3B-day3 Control medium 300dpi.tif]

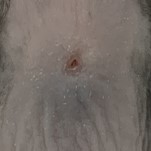

Supplement: S1 Raw data — (ZIP) [file pone.0317766.s001.zip › Archive/3B-day12 tlr2and6 hmc-1cm300.jpg]

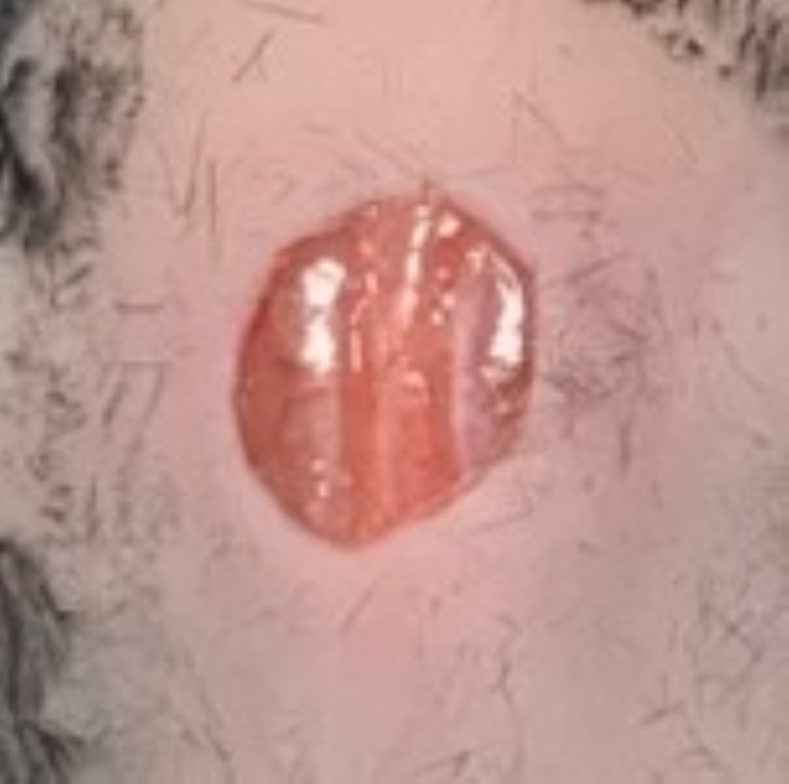

Supplement: S1 Raw data — (ZIP) [file pone.0317766.s001.zip › Archive/3B-day1 HMC-1 CM 300dpi.tif]

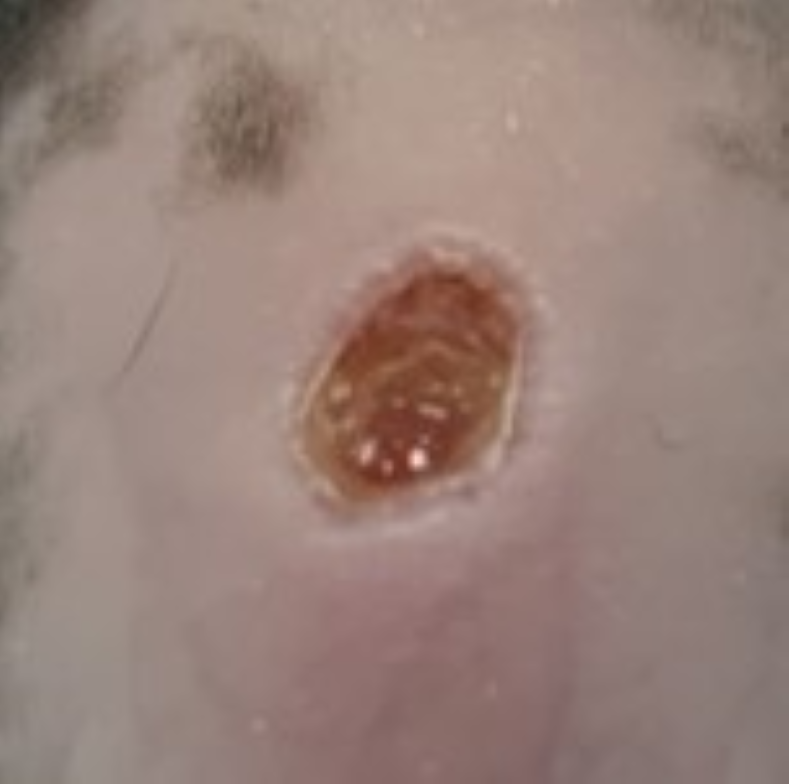

Supplement: S1 Raw data — (ZIP) [file pone.0317766.s001.zip › Archive/3B-day6 Control medium 300dpi.tif]

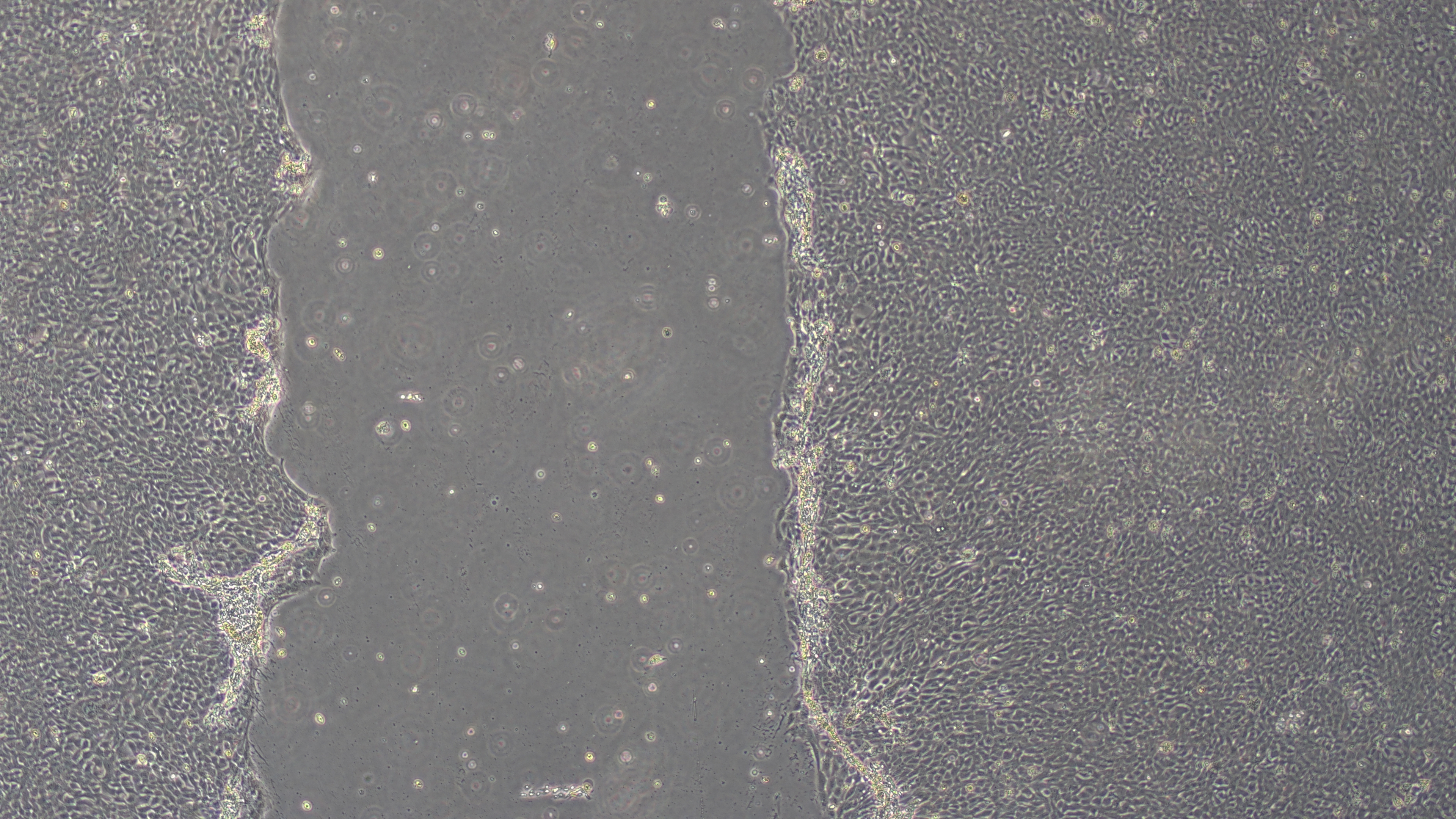

Supplement: S1 Raw data — (ZIP) [file pone.0317766.s001.zip › Archive/1A-H48 h.jpg]

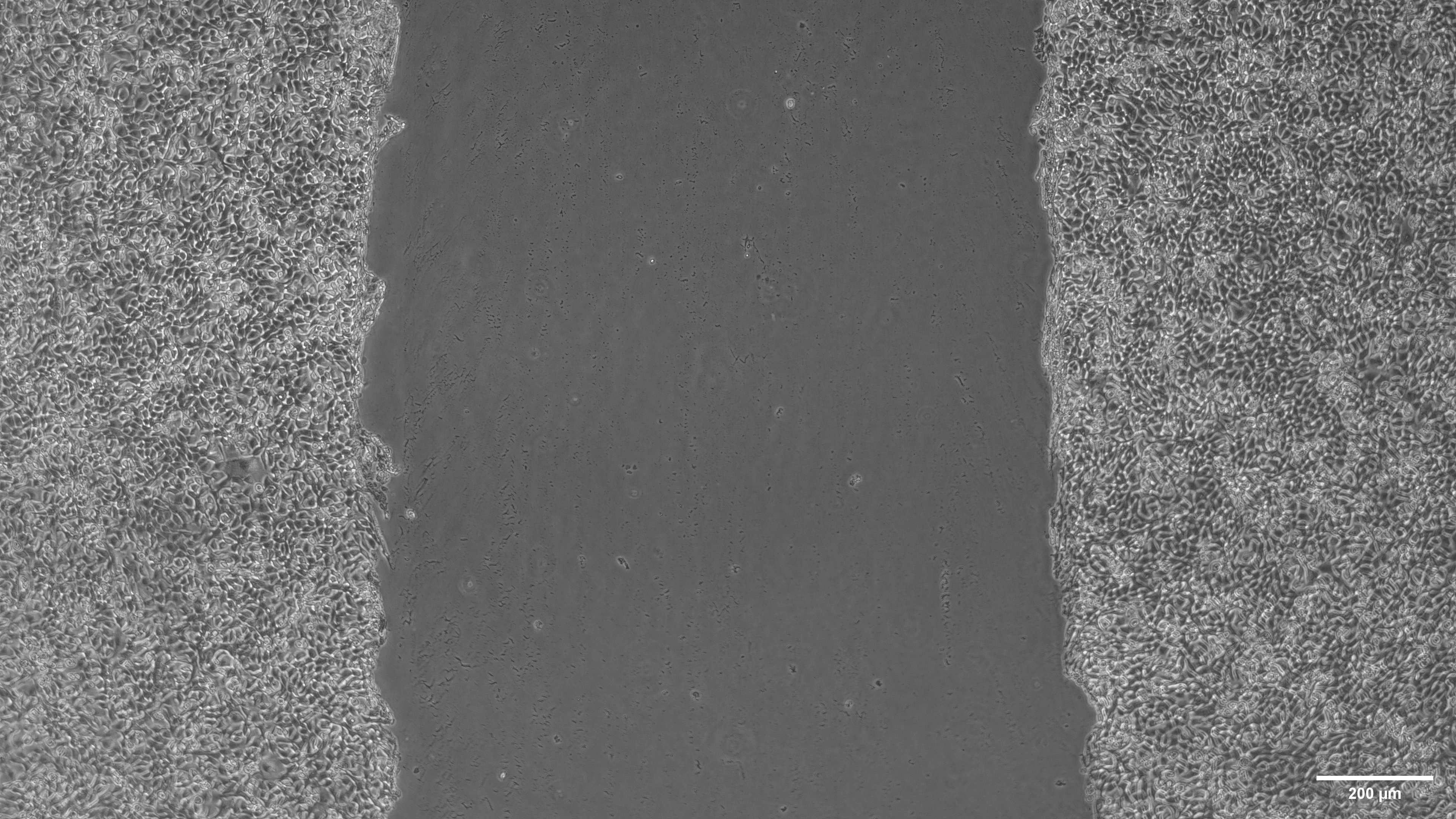

Supplement: S1 Raw data — (ZIP) [file pone.0317766.s001.zip › Archive/5B-FH0.jpg]

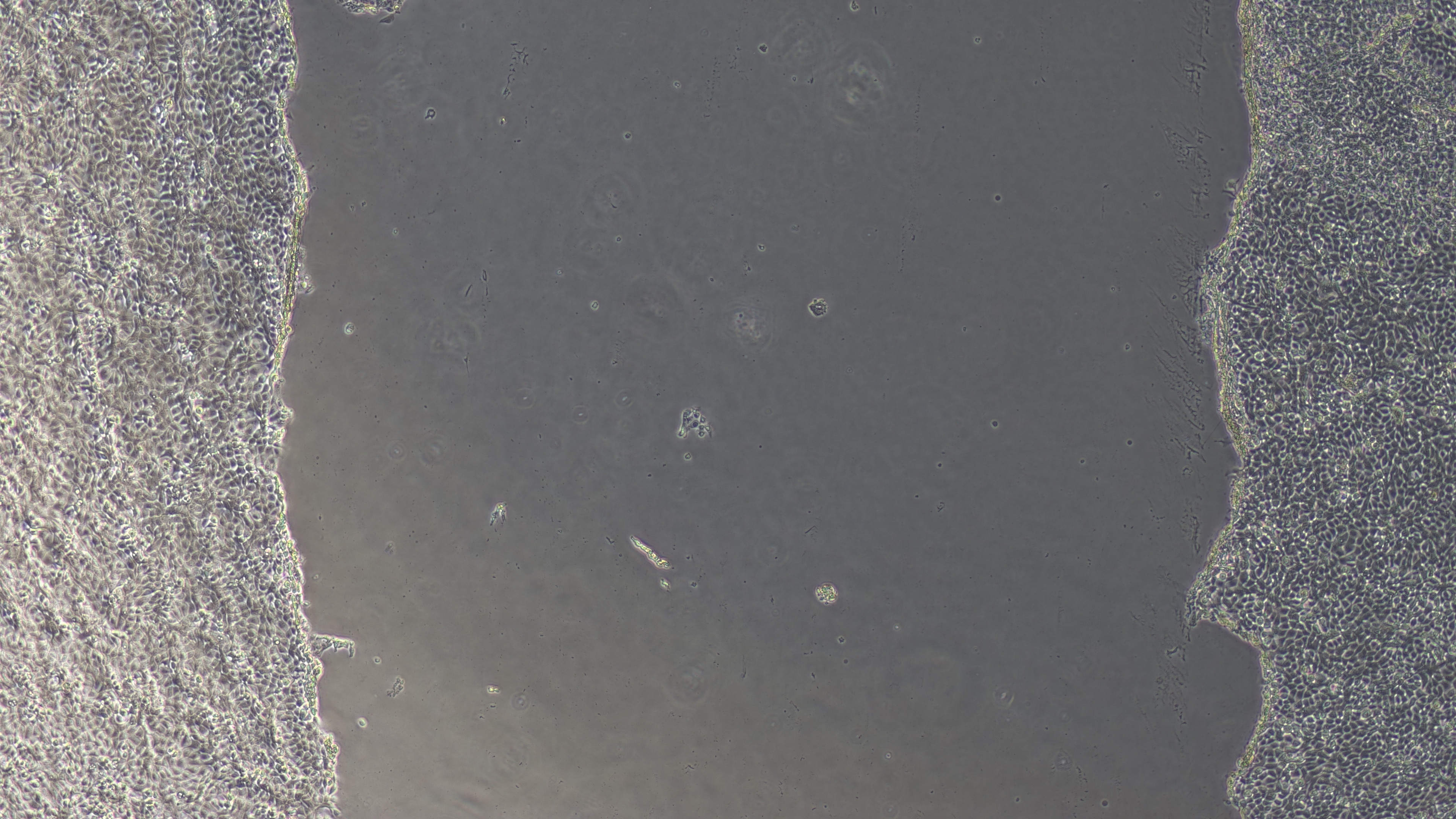

Supplement: S1 Raw data — (ZIP) [file pone.0317766.s001.zip › Archive/1A-mito0 h.jpg]

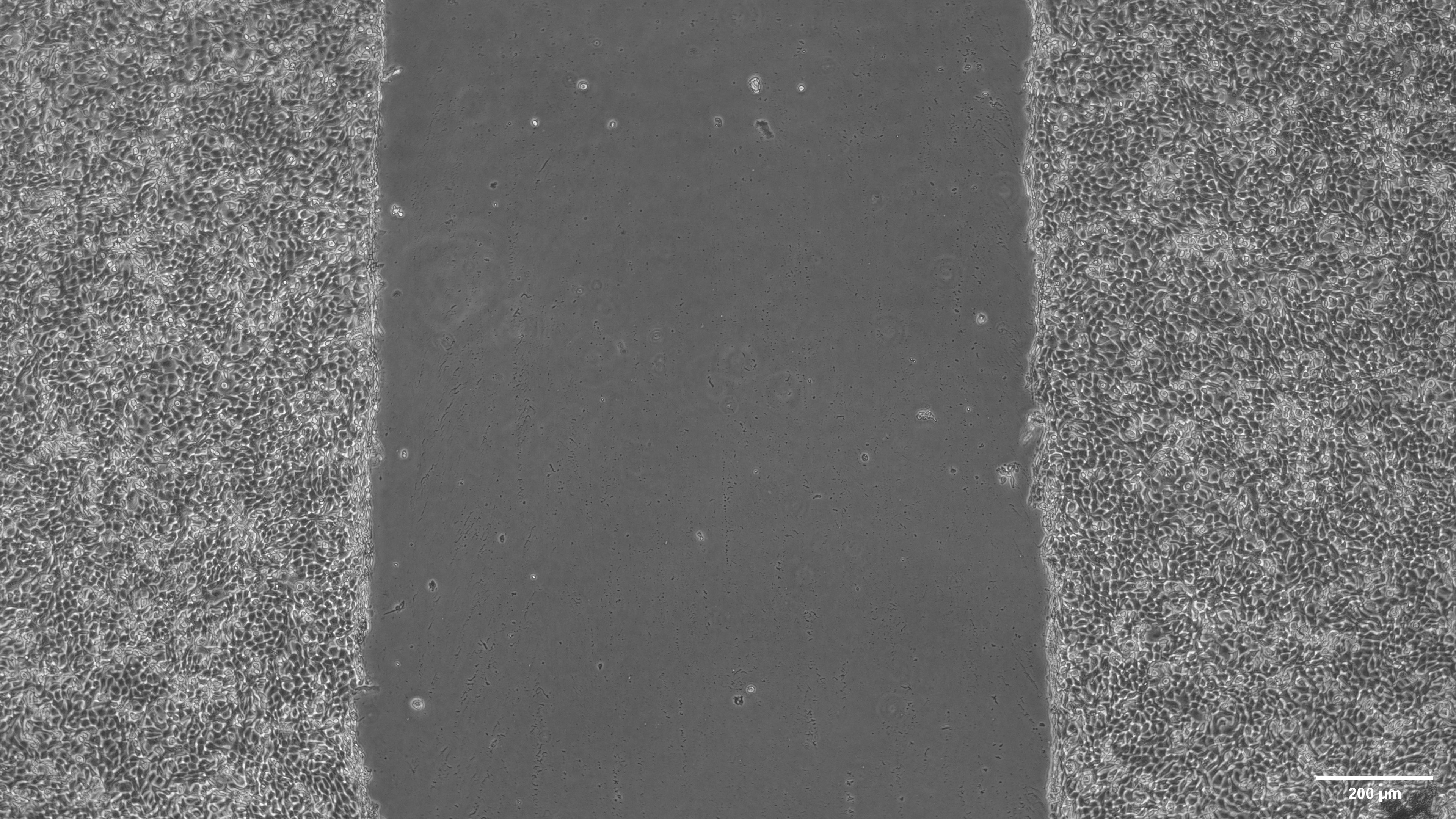

Supplement: S1 Raw data — (ZIP) [file pone.0317766.s001.zip › Archive/5B-FHA0.jpg]

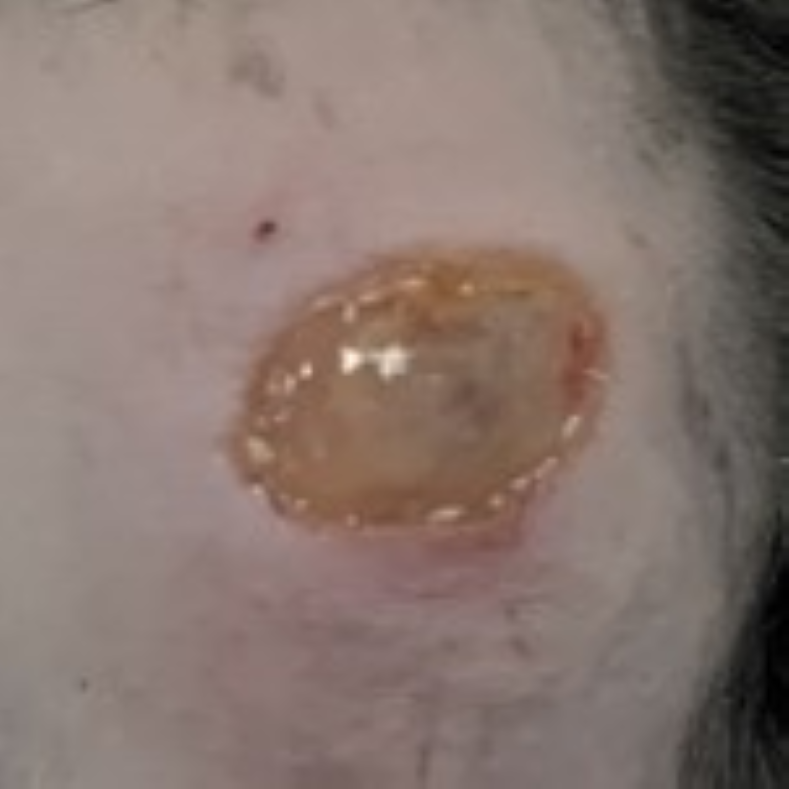

Supplement: S1 Raw data — (ZIP) [file pone.0317766.s001.zip › Archive/3B-day3 FSL-1 300dpi.tif]

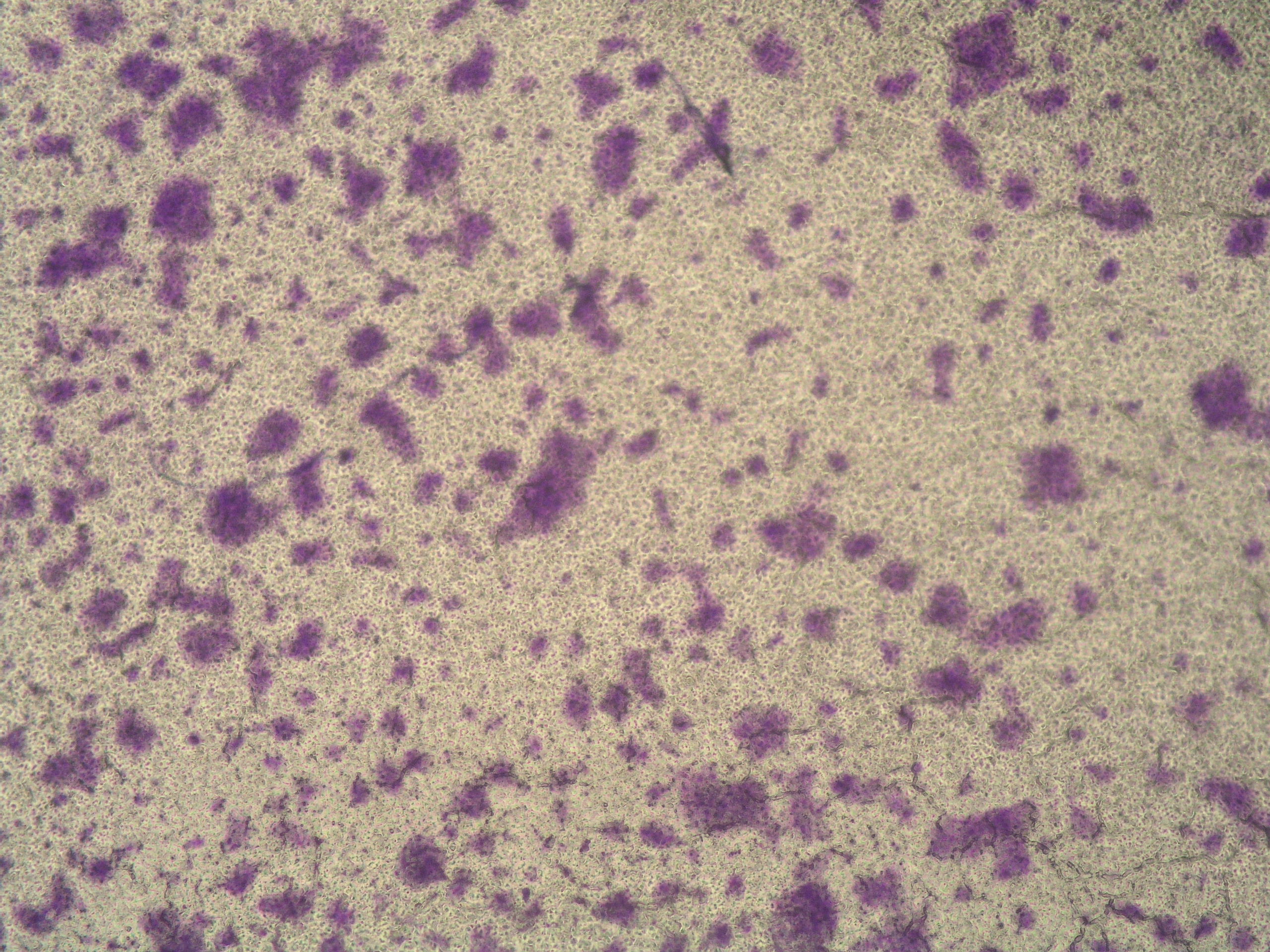

Supplement: S1 Raw data — (ZIP) [file pone.0317766.s001.zip › Archive/1C-Control medium 24h (300dpi).JPG]

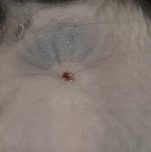

Supplement: S1 Raw data — (ZIP) [file pone.0317766.s001.zip › Archive/3B-day12 hmc-1 cm300.jpg]

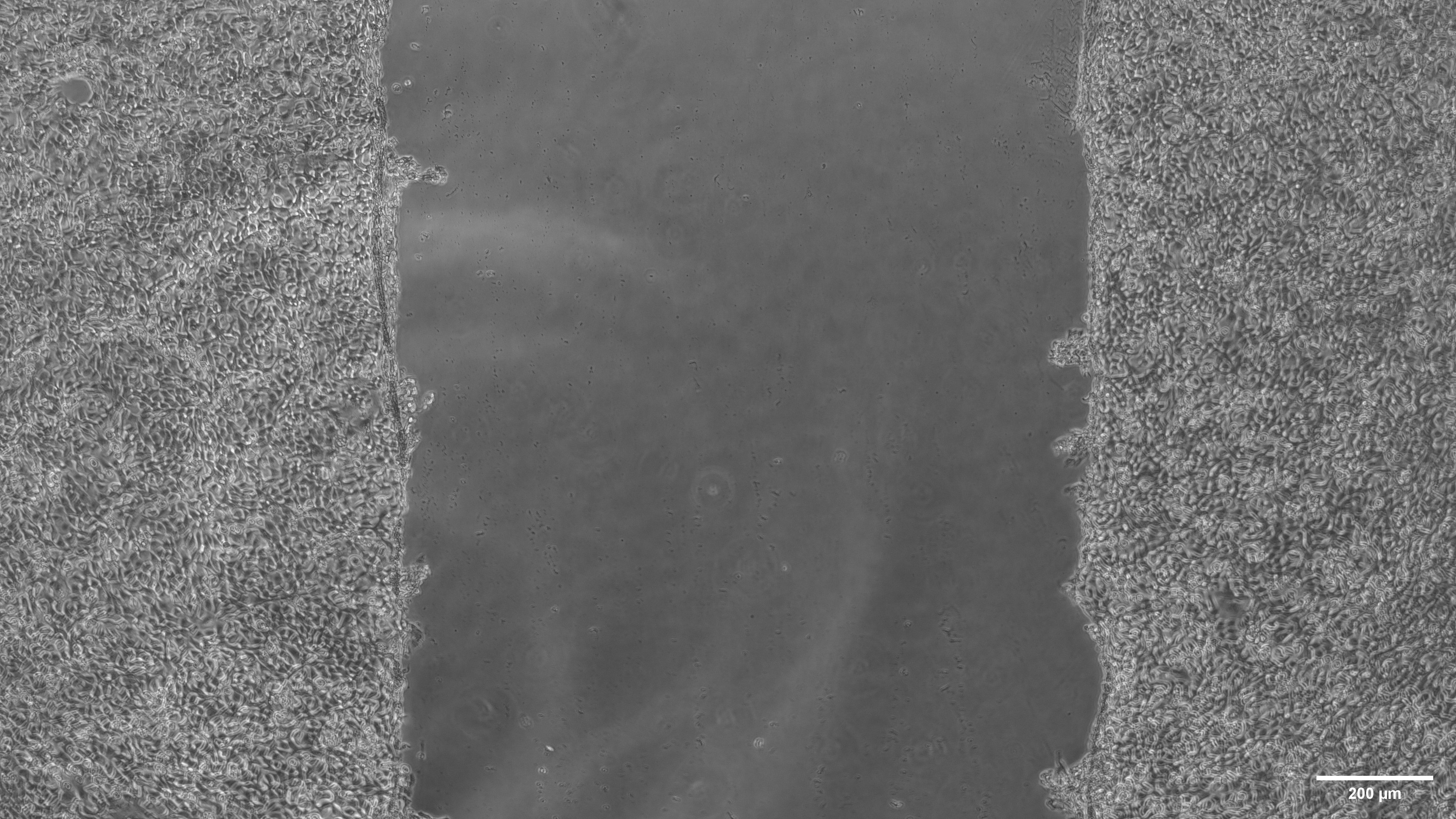

Supplement: S1 Raw data — (ZIP) [file pone.0317766.s001.zip › Archive/5B-C0.jpg]

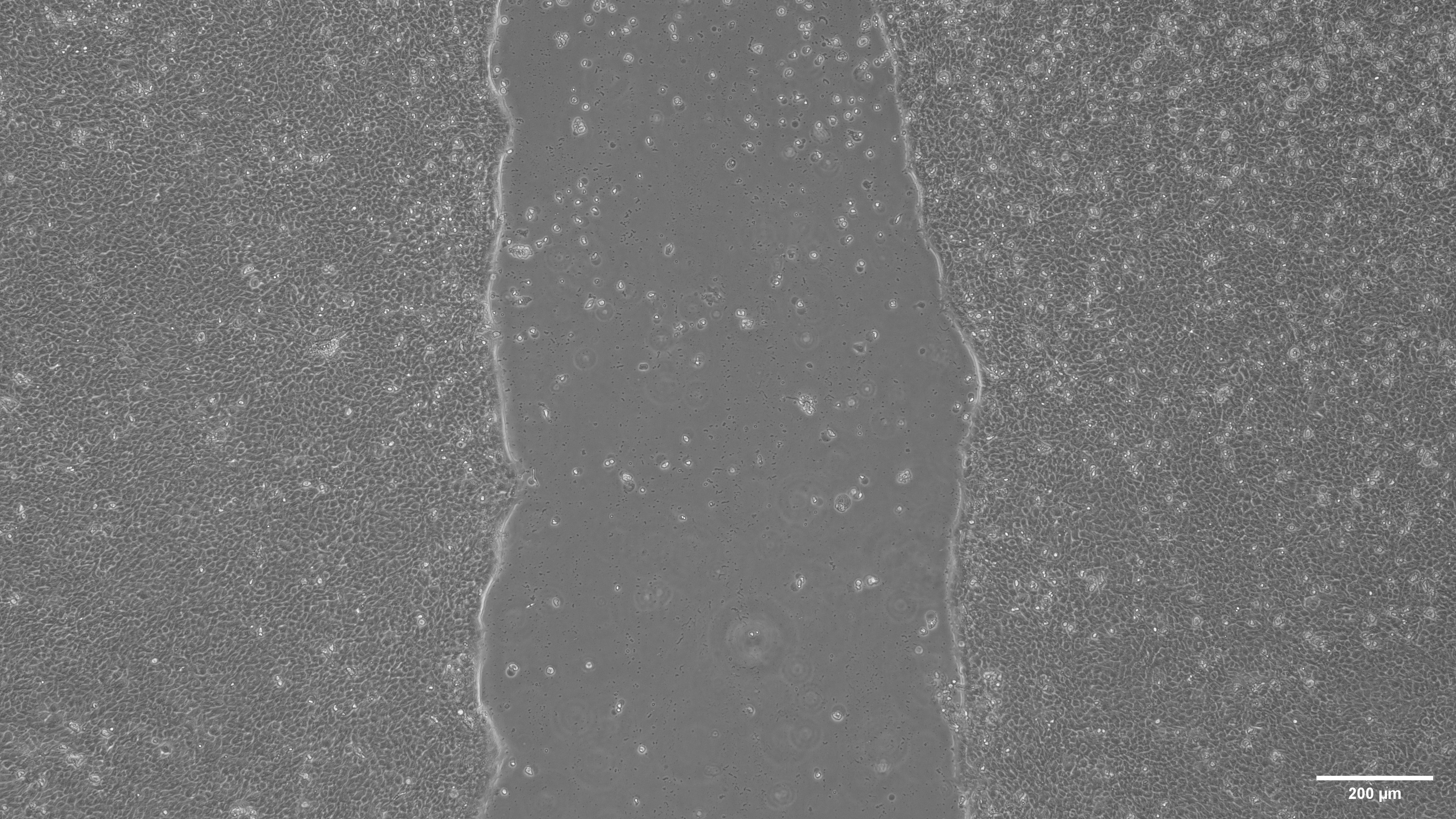

Supplement: S1 Raw data — (ZIP) [file pone.0317766.s001.zip › Archive/5B-HA24.jpg]

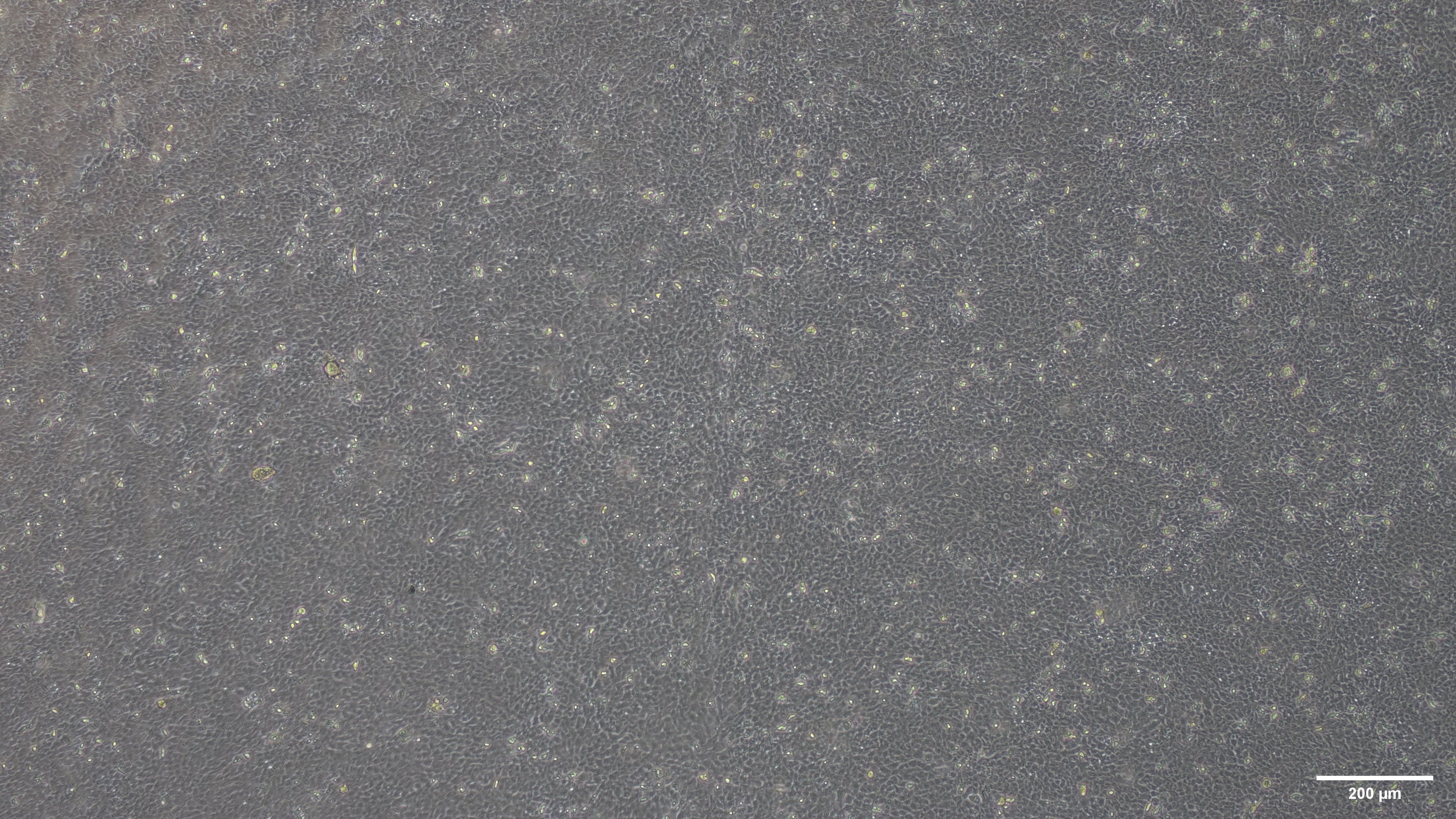

Supplement: S1 Raw data — (ZIP) [file pone.0317766.s001.zip › Archive/5B-FH48.jpg]

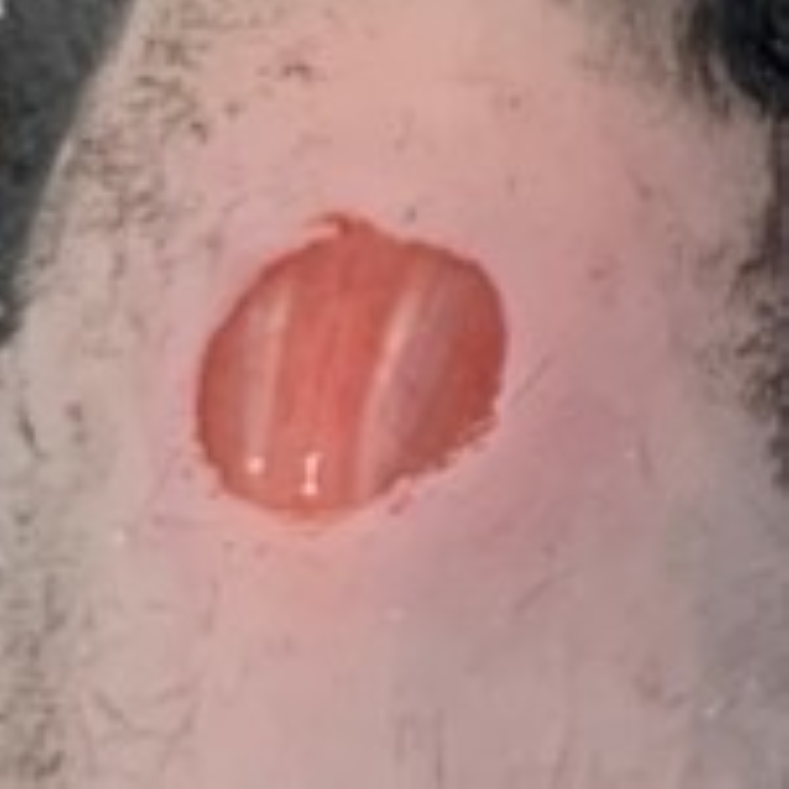

Supplement: S1 Raw data — (ZIP) [file pone.0317766.s001.zip › Archive/3B-day1 Control medium 300dpi.tif]
